# Supplementary figures and images for: Specificity of Loxosceles α clade phospholipase D enzymes for choline-containing lipids: Role of a conserved aromatic cage
Source: PLoS Comput Biol. 2022 Feb 18;18(2):e1009871. doi: 10.1371/journal.pcbi.1009871 (PMC8893692; doi:10.1371/journal.pcbi.1009871)

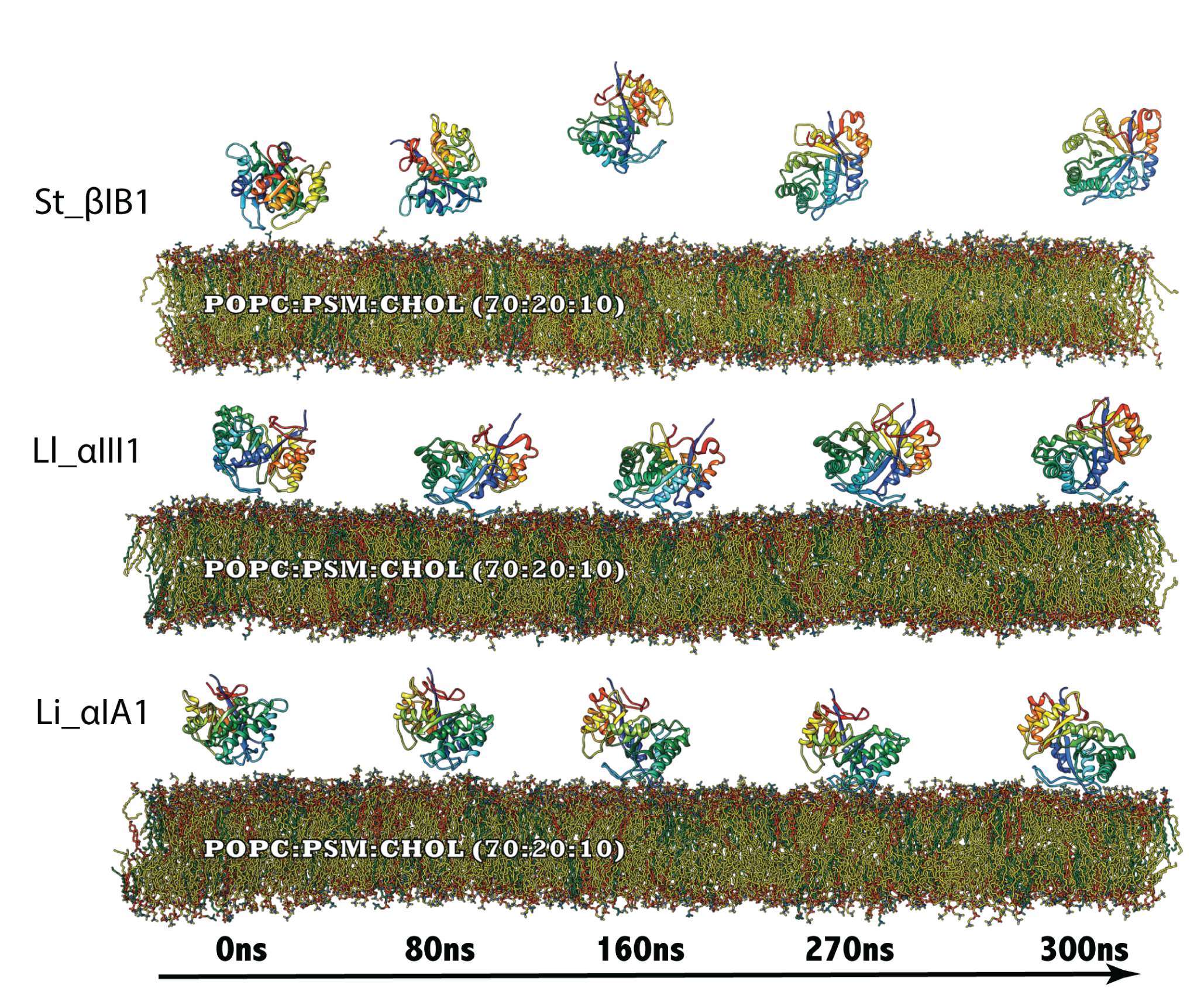

Supplement: S1 Fig — Snapshots at 0, 80, 160, 270 and 300 ns were extracted to follow the evolution of the systems during the simulations. (TIF) [file pcbi.1009871.s001.tif]

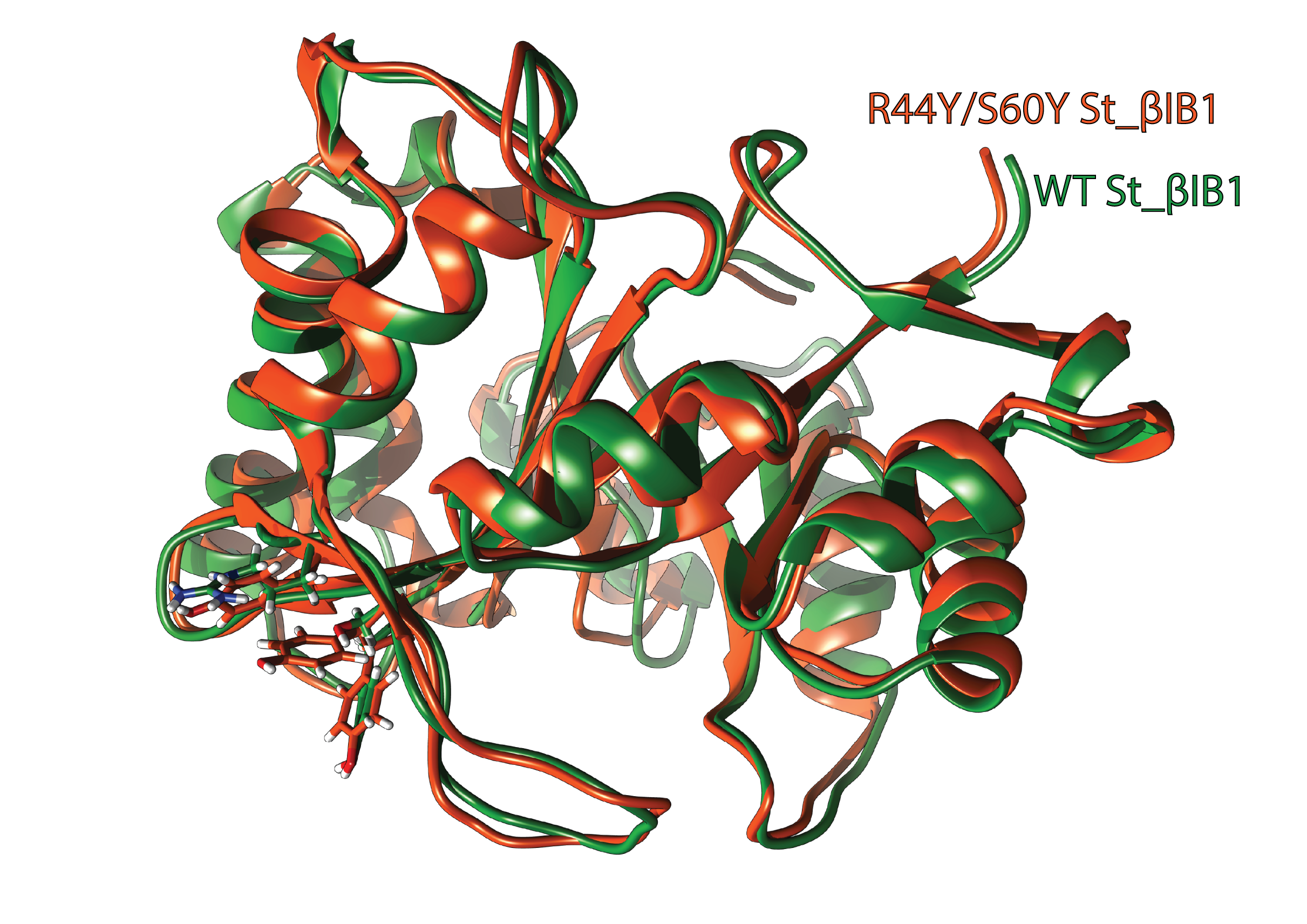

Supplement: S2 Fig — The structures used for the alignment are final frames of each production run of WT St_βIB1 and R44Y/S60Y St_βIB1 each on a pure POPC bilayer. The calculated RMSD of the protein backbone is 1 Å in both cases. (TIF) [file pcbi.1009871.s002.tif]

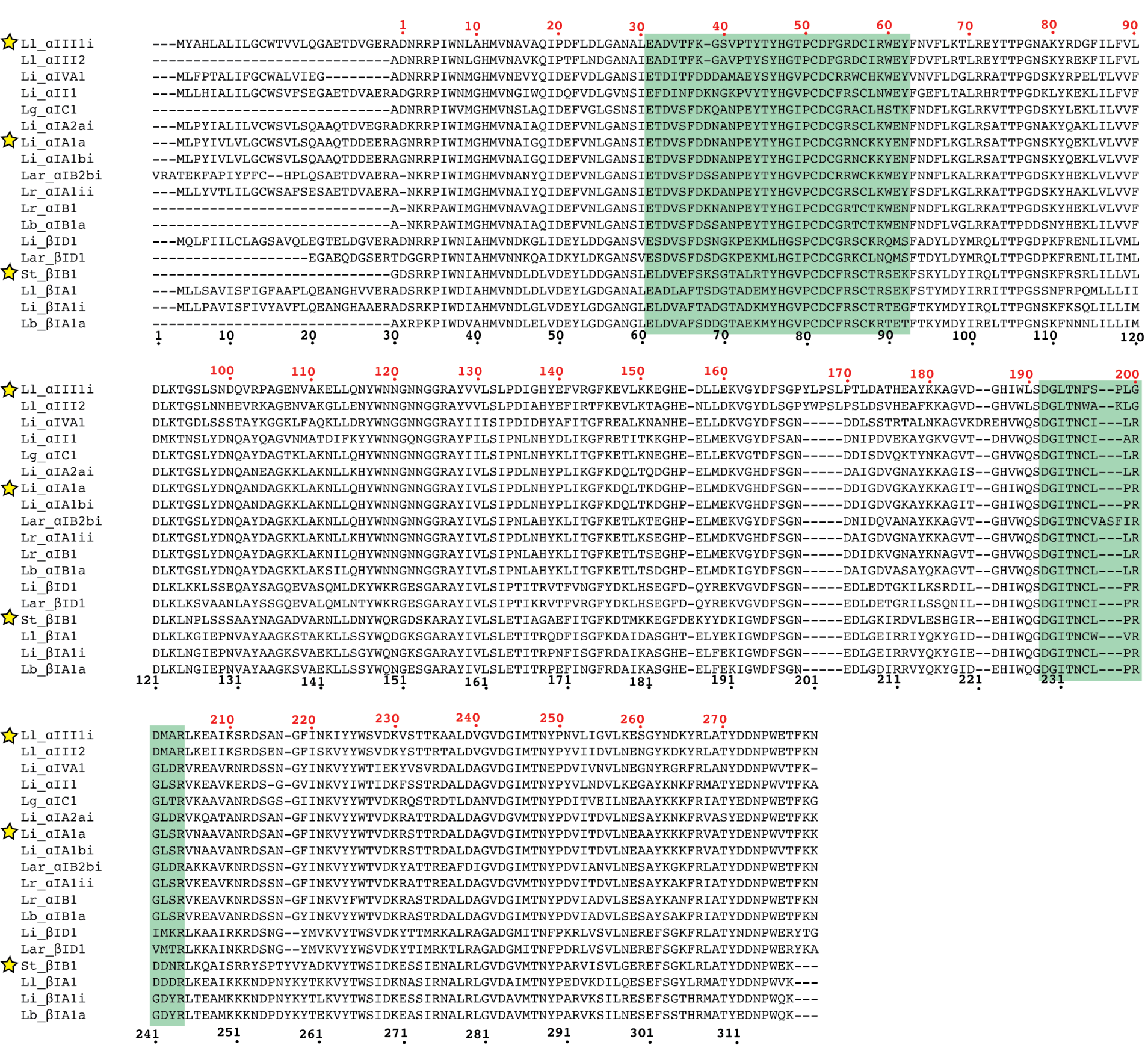

Supplement: S3 Fig — The Uniprot identifiers of the aligned sequences are provided in the Methods section of the article. The green background highlights the catalytic and the flexible loops. The proteins simulated in this study are indicated with a yellow star. The red numbering corresponds to the numbering of the St_βIB1 structure (PDBid: 4Q6X). The black numbering indicates the position in the multiple sequence alignment. (TIF) [file pcbi.1009871.s003.tif]
