# Supplementary material for: Specificity of Loxosceles α clade phospholipase D enzymes for choline-containing lipids: Role of a conserved aromatic cage
Source: PLoS Comput Biol. 2022 Feb 18;18(2):e1009871. doi: 10.1371/journal.pcbi.1009871 (PMC8893692; doi:10.1371/journal.pcbi.1009871)
Supplement: S2 Text — (PDF) [file pcbi.1009871.s005.pdf]

Depth of Anchoring

Table A. Anchoring depth of Li_αIA1, Ll_αIII1 and R44Y/S60Y St_βIB1 on a pure POPC bilayer. The depth represents the distance between the C-alpha atom of each residue and the upper phosphate plane. R1 and R2 stand for replica 1 and replica 2. Only residues with a relevant anchoring depth are indicated in this table.

SSE	Residue	Depth (Å)					
		Li_αIA1		Ll_αIII1		R44Y/S60Y St_βIB1	
		R1	R2	R1	R2	R1	R2
β2α2	37	5.1	6.6	4.5	4.1	2.2	4.2
	38	2.4	3.9	1.3	0.6	0.8	2.6
	39	2.4	4.6	2.3	1.3	1.7	2.3
	44	5.4	6.7	3.6	3.2	5.9	4.4
	46	5.4	6.4	3.7	3.8	6.1	5.2
	48	1.6	2.3	0.4	0.8	6.1	2.6
	49	-0.9	-0.1	-0.2	0.3	0.6	0.9
	50	-0.5	0.2	0.9	1.7	-1.0	0.3
	51	2.9	3.7	3.4	3.6	1.8	3.9
	52	4.9	6.0	5.7	5.0	3.6	6.1
	53	3.4	5.1	4.4	3.5	3.5	8.5
	54	4.1	5.9	5.1	5.7	0.4	7.1
	55	4.8	5.5	4.5	5.6	1.8	8.1
	56	2.7	3.2	1.7	3.0	2.3	6.8
	57	2.2	2.6	2.2	2.5	4.7	6.0
	58	1.5	1.8	-0.8	-0.4	2.5	3.5
	59	3.8	5.0	0.9	1.6	4.1	5.7
	60	3.8	5.8	1.9	2.4	5.3	5.3
β3α3	95	2.5	5.1	4.8	4.9	5.2	2.1
	96	3.8	6.6	2.5	1.4	2.0	1.7
	97	3.9	7.1	4.9	2.3	3.7	2.6
	98	3.8	7.5	5.4	1.5	3.1	4.1
β6α6	201	4.2	7.8	10.0	12.3	11.5	15.1
	202	4.4	7.9	12.8	15.3	13.5	15.6

Table B. Anchoring depth of Li_αIA1 and Ll_αIII1 on a POPC:PSM:CHOL (70:20:10) bilayer. The depth represents the distance between the C-alpha atom of each residue and the upper phosphate plane. R1 and R2 stand for replica 1 and replica 2. Only residues with a relevant anchoring depth are indicated in this table.

SSE	Residue	Depth (Å)			
		**Li_αIA1**		**Ll_αIII1**	
		**R1**	**R2**	**R1**	**R2**
β2α2	37	7.4	7.0	7.2	5.3
	38	5.2	5.1	4.7	2.4
	39	4.8	4.2	5.2	2.1
	44	9.2	9.5	6.3	5.5
	46	7.5	7.0	4.5	4.9
	48	2.1	1.9	2.4	1.3
	49	-0.4	-0.5	0.2	-0.3
	50	-0.1	-0.5	0.9	0.5
	51	3.5	2.3	4.1	3.8
	52	5.3	4.4	5.8	5.7
	53	4.0	3.4	3.9	4.5
	54	5.4	4.7	5.0	6.3
	55	6.3	5.4	5.5	5.9
	56	5.0	3.7	3.2	3.0
	57	4.2	4.1	2.4	2.2
	58	3.7	2.3	0.6	0.4
	59	7.4	5.5	3.7	3.1
	60	8.5	7.0	5.2	4.2
β3α3	95	3.0	3.0	6.3	3.2
	96	1.7	2.1	4.1	1.7
	97	3.4	3.6	6.4	3.8
	98	3.4	4.1	6.3	2.6
β6α6	201	3.3	3.0	8.8	8.1
	202	3.4	4.8	7.4	10.7

Table C. Anchoring depth of St_βIB1 on a POPC:POPE (50:50) bilayer. The depth represents the distance between the C-alpha atom of each residue and the upper phosphate plane. R1 and R2 stand for replica 1 and replica 2. Only residues with a relevant anchoring depth are indicated in this table.

SSE	residue	Depth	
		R1	R2
β2α2	H47	10.7	11.0
	G48	10.0	10.0
	V49	6.8	7.9
	P50	4.4	6.7
	C51	4.4	5.1
	D52	3.7	4.5
	C53	0.0	0.9
	F54	-1.8	-1.3
	R55	0.9	1.2
	S56	2.3	2.5
	C57	4.9	4.7
	T58	6.4	5.7
	R59	9.7	7.7
β6	D192	11.9	13.6
	G193	8.7	10.5
	I194	6.0	7.7
β6α6	T195	4.2	5.4
	N196	2.7	3.2
	C197	-0.9	-0.2
	L198	-1.0	-0.6
	P199	0.8	0.6
	R200	3.8	5.0
	D201	4.3	6.3
	D202	7.4	9.2
α6	N203	8.6	11.2
	R204	11.7	13.7
β7α7	W226	10.7	11.0
	S227	8.6	8.5
	I228	6.4	6.1
	D229	5.5	3.4
	K230	2.8	1.9
α7	E231	4.3	3.2
	S232	2.8	2.5
	S233	4.3	4.4
	I234	7.6	7.4
	E235	7.7	7.1
	N236	7.0	7.5
	A237	9.9	10.6
