# Supplementary material for: Specificity of Loxosceles α clade phospholipase D enzymes for choline-containing lipids: Role of a conserved aromatic cage
Source: PLoS Comput Biol. 2022 Feb 18;18(2):e1009871. doi: 10.1371/journal.pcbi.1009871 (PMC8893692; doi:10.1371/journal.pcbi.1009871)
Supplement: S3 Text — (PDF) [file pcbi.1009871.s006.pdf]

Liposome binding assay data

Table A. Data from three individual experiments for wild-type St_βIB1 and the R44Y/S60Y variant binding to 1:1 sphingomyelin:cholesterol liposomes. Numbers indicate the percentage of protein found in the pellet fraction. Note that despite some run-to-run variation in absolute numbers, the R44Y/S60Y variant shows consistently higher levels of protein pelleted by the liposomes, relative to the wild type. Details of experimental conditions and analysis are described in Methods.

	- Liposomes	+ Liposomes	Difference	R44Y/S60Y minus wild-type
Wild type run 1	10	33	23	
R44Y/S60Y run 1	7	43	36	13
Wild type run 2	16	26	10	
R44Y/S60Y run 2	7	34	27	17
Wild type run 3	10	22	12	
R44Y/S60Y run 3	8	30	22	10
