# Supplementary material for: Specificity of Loxosceles α clade phospholipase D enzymes for choline-containing lipids: Role of a conserved aromatic cage
Source: PLoS Comput Biol. 2022 Feb 18;18(2):e1009871. doi: 10.1371/journal.pcbi.1009871 (PMC8893692; doi:10.1371/journal.pcbi.1009871)
Supplement: S4 Text — (PDF) [file pcbi.1009871.s007.pdf]

Conformations of POPC headgroups

Table A. Average angles between P-N vectors and the membrane normal for POPC lipids bound or unbound to the aromatic cage.

System	Number of “free” PC lipids	Unbound (degrees)		Bound (degrees)	
		R1	R2	R1	R2
1	255	63 ± 20	71 ± 25	50 ± 20	60 ± 24
2	255	67 ± 20	70 ± 26	55 ± 17	62 ± 28
5	179	70 ± 24	69 ± 28	61 ± 22	63 ± 23
6	179	74 ± 28	75 ± 29	68 ± 26	71 ± 20
9	127	-	68 ± 25	-	62 ± 30
