# Supplementary material for: Specificity of Loxosceles α clade phospholipase D enzymes for choline-containing lipids: Role of a conserved aromatic cage
Source: PLoS Comput Biol. 2022 Feb 18;18(2):e1009871. doi: 10.1371/journal.pcbi.1009871 (PMC8893692; doi:10.1371/journal.pcbi.1009871)

Stability of the Molecular Dynamics simulations

Figure A. Evolution of the protein backbone Root Mean Square Deviation (RMSD) during all simulations involving a pure POPC bilayer. Replica 1 is represented in orange and replica 2 in cyan.

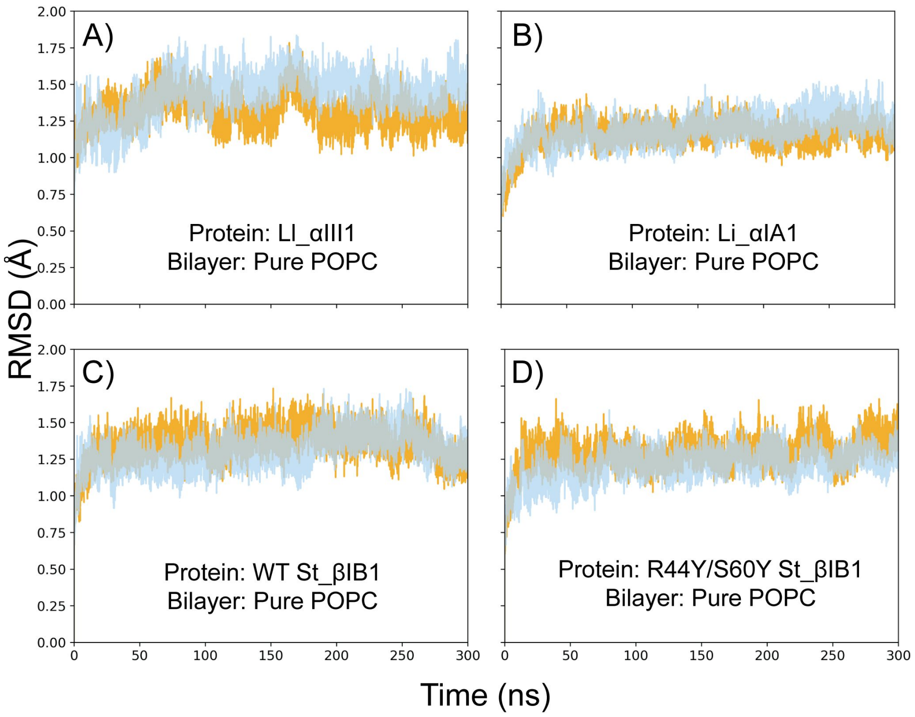

Figure B. Evolution of the protein backbone Root Mean Square Deviation (RMSD) during all simulations involving a PC:SM:CHOL (70:20:10) or a POPC:POPE (50:50). Replica 1 is represented in orange and replica 2 in cyan.

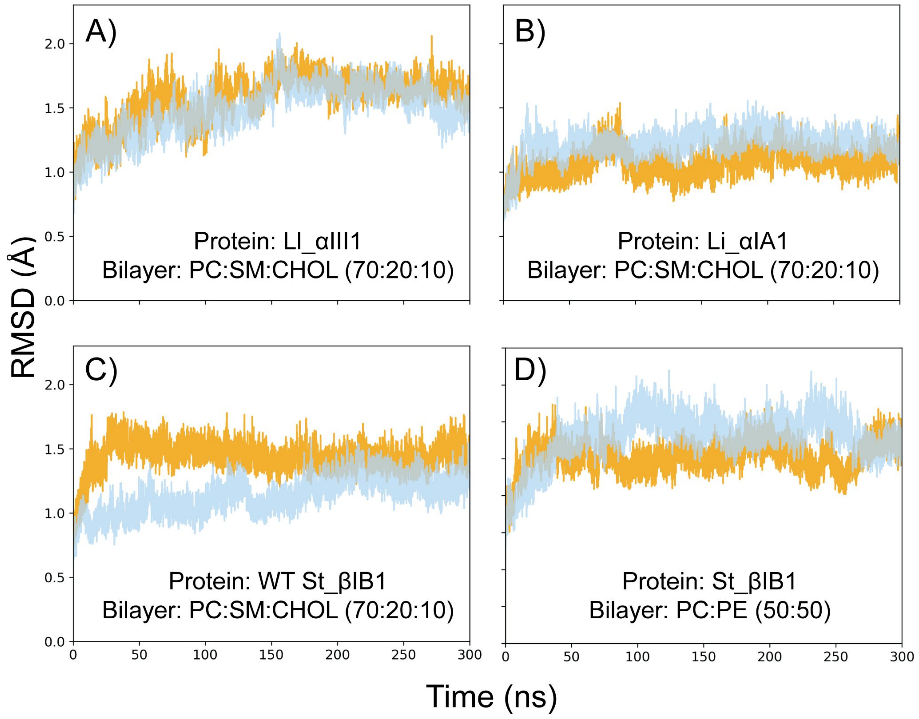

Figure C. Evolution of the protein backbone Root Mean Square Deviation (RMSD) during the simulations of Li_αIA1 on a POPC:POPE (50:50) bilayer. Replica 1 is represented in orange and replica 2 in cyan.

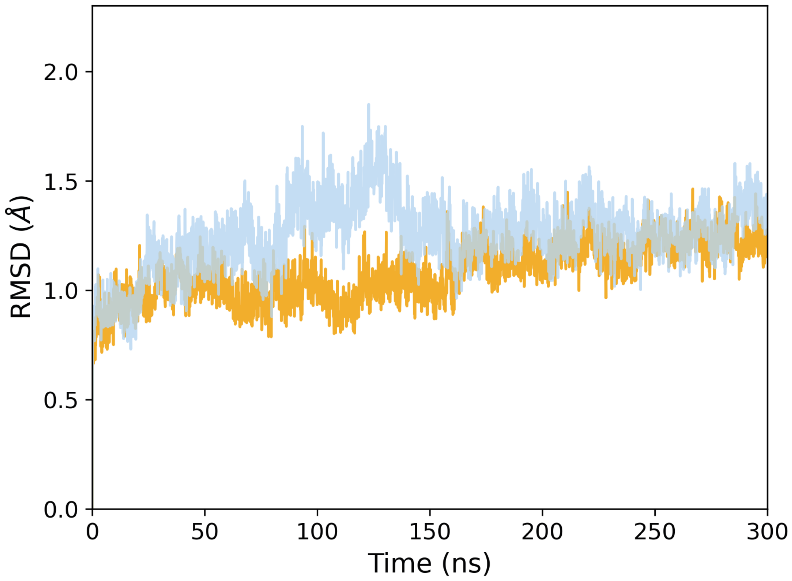

Figure D. Simulations with POPC bilayers: simulation box area in the bilayer plane. Replica 1 is represented in orange and replica 2 in cyan.

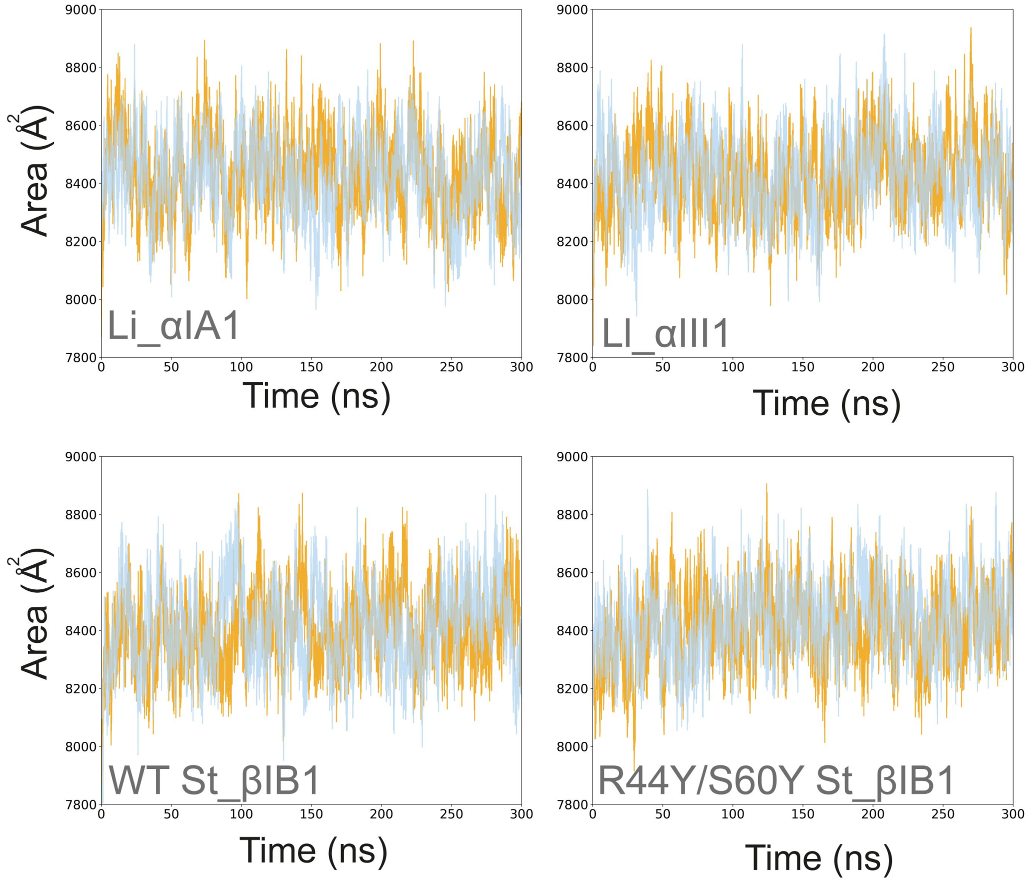

Figure E. Simulations with PC:SM:CHOL (70:20:10) bilayers: simulation box area in the bilayer plane. Replica 1 is represented in orange and replica 2 in cyan.

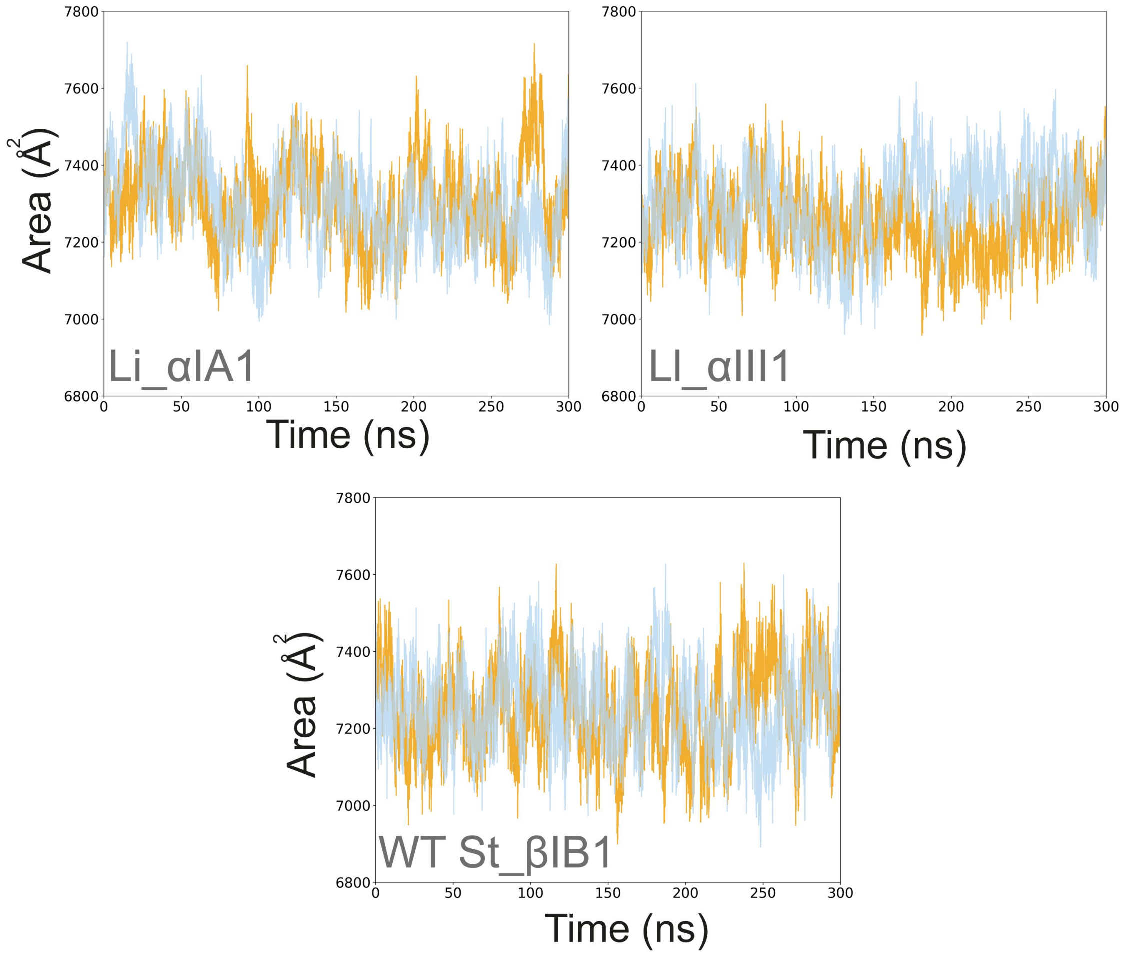

Figure F. Simulations with POPC:POPE (50:50) bilayers: simulation box area in the bilayer plane. Replica 1 is represented in orange and replica 2 in cyan.

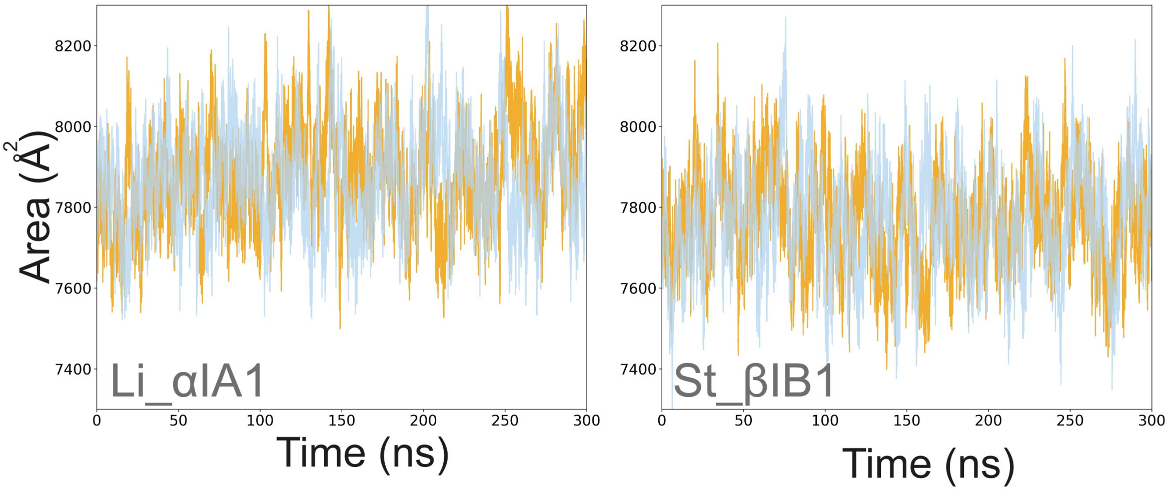

Figure G. Minimum distance between PLDs and their nearest image in simulations with POPC bilayers. Replica 1 is represented in orange and replica 2 in cyan.

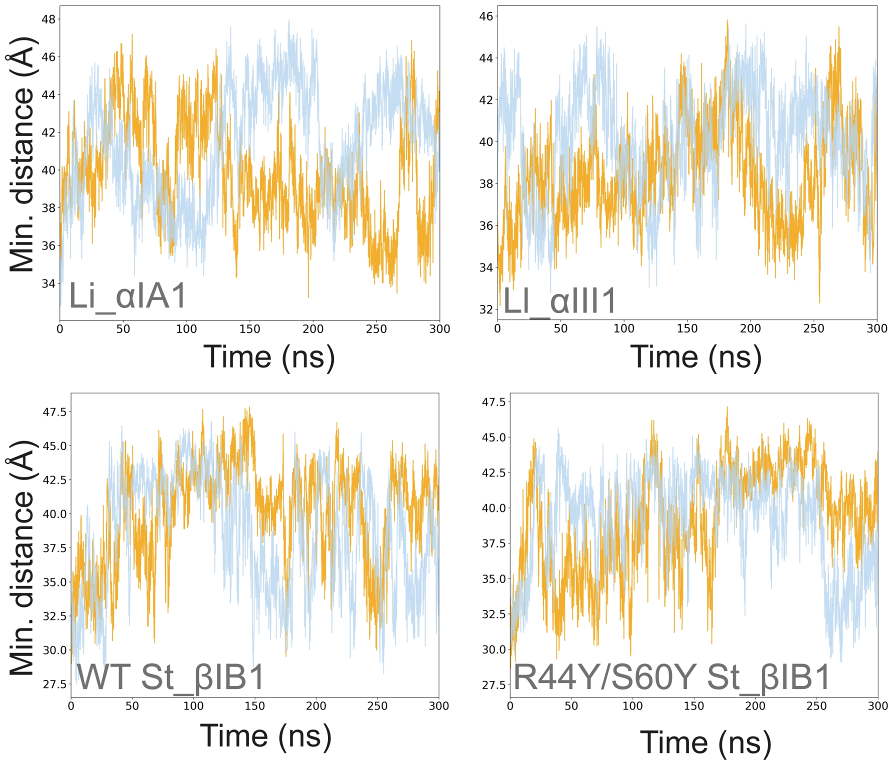

Figure H. Minimum distance between PLDs and their nearest image in simulations with PC:SM:CHOL (70:20:10) bilayers. Replica 1 is represented in orange and replica 2 in cyan.

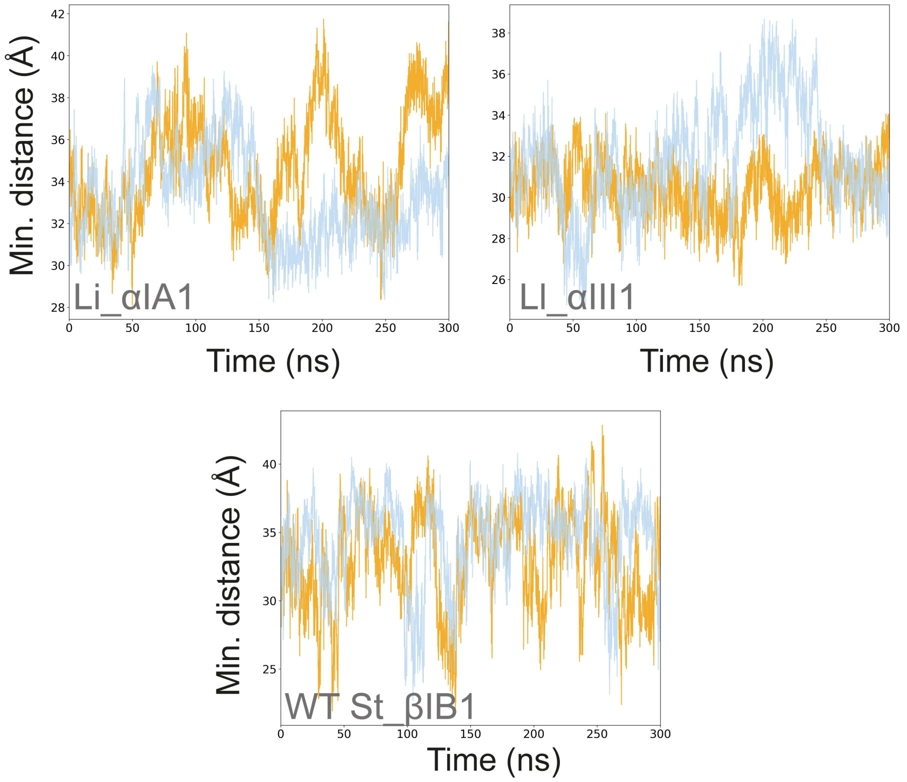

Figure I. Minimum distance between PLDs and their nearest image in simulations with PC:PE (50:50) bilayers. Replica 1 is represented in orange and replica 2 in cyan.

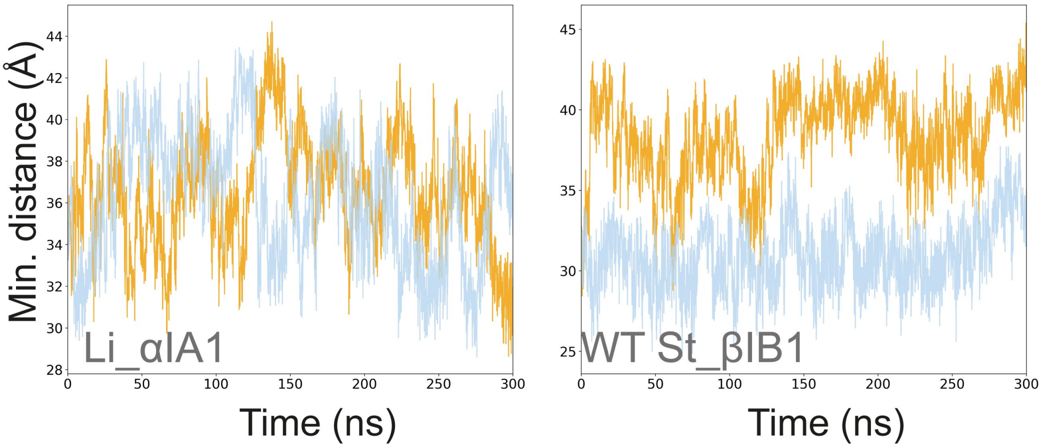

Supplement: S5 Text — (PDF) [file pcbi.1009871.s008.pdf]
