# Supplementary material for: Specificity of Loxosceles α clade phospholipase D enzymes for choline-containing lipids: Role of a conserved aromatic cage
Source: PLoS Comput Biol. 2022 Feb 18;18(2):e1009871. doi: 10.1371/journal.pcbi.1009871 (PMC8893692; doi:10.1371/journal.pcbi.1009871)

Proteins-bilayers distances during the Molecular Dynamics simulations

Figure A. Minimum protein-bilayer distance in simulations of Li_αIA1, Ll_αIII1 and WT St_βIB1 with a PC:SM:CHOL (70:20:10) bilayer. Replica 1 is represented in orange and replica 2 in cyan.

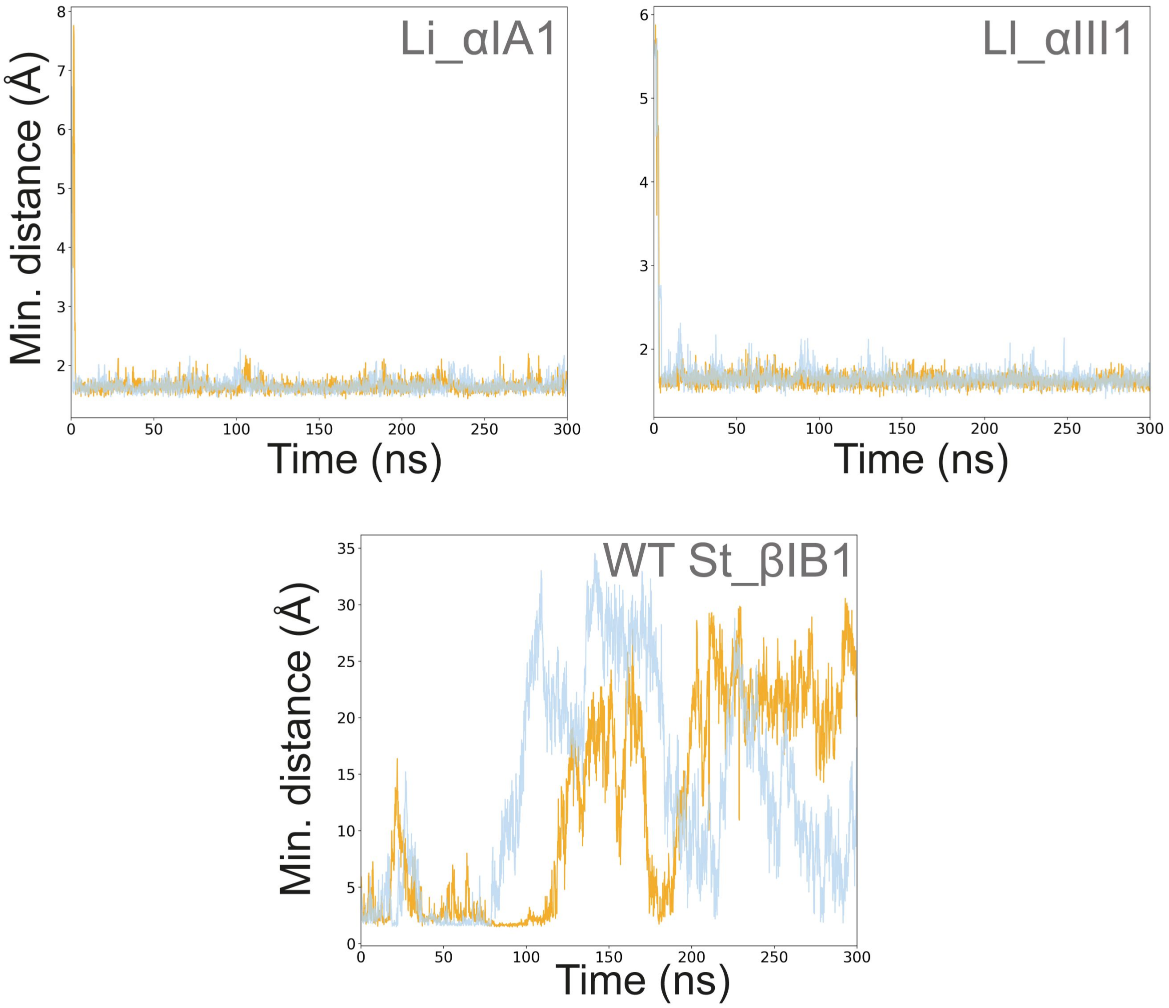

Figure B. Minimum protein-bilayer distance in simulations of Li_αIA1, Ll_αIII1, WT St_βIB1 and R44Y/S60Y with a POPC bilayer. Replica 1 is represented in orange and replica 2 in cyan.

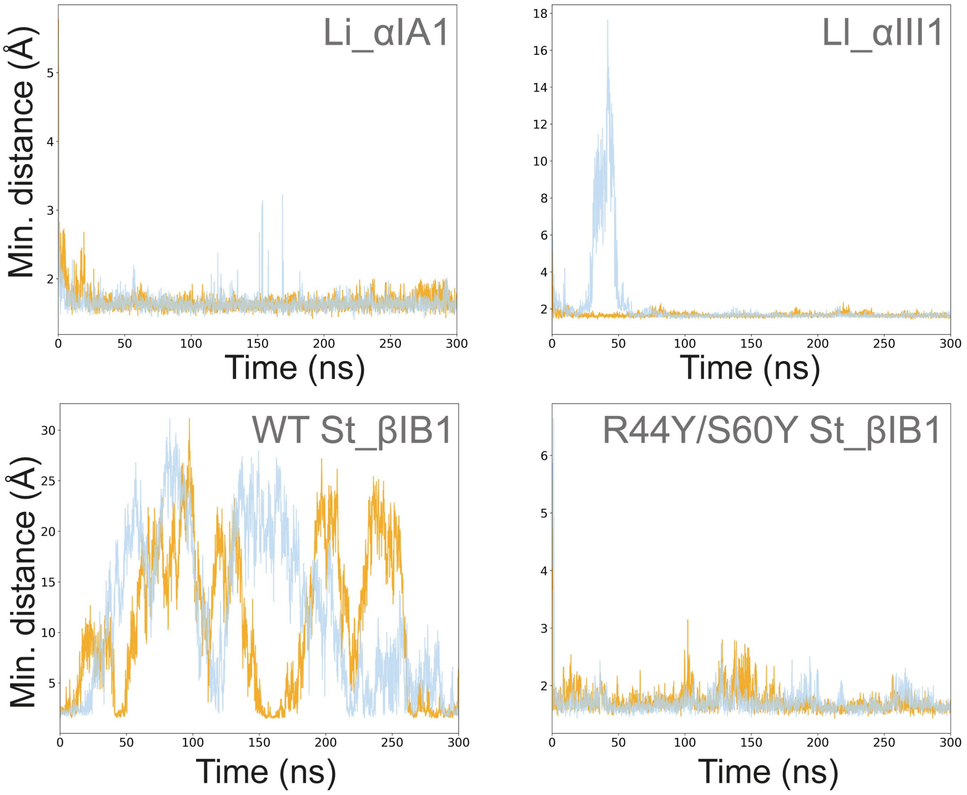

Figure C. Minimum protein-bilayer distance in simulations of Li_αIA1 and WT St_βIB1 with a POPC:POPE (50:50) bilayer. Replica 1 is represented in orange and replica 2 in cyan.

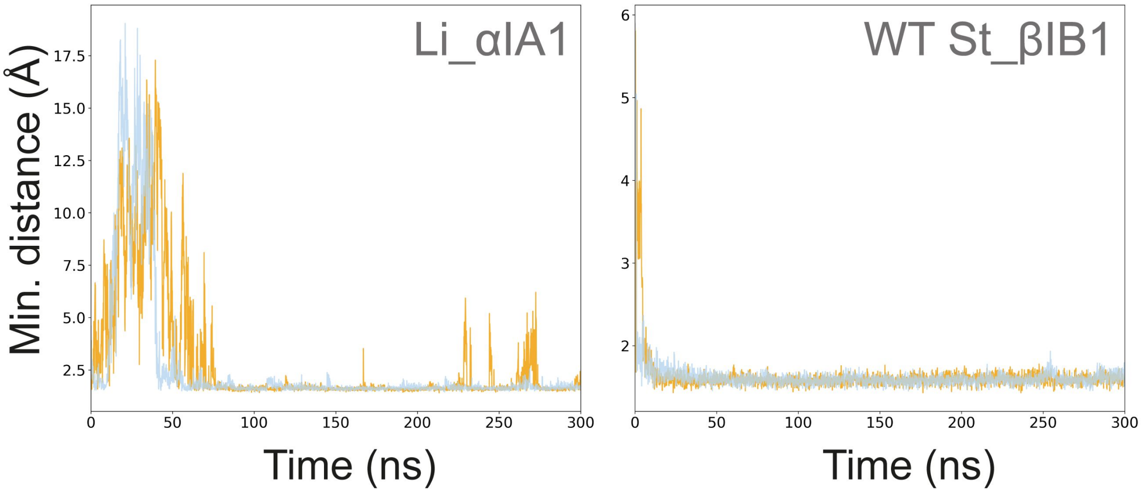

Figure D. Minimum protein-bilayer distance in the 1 µs-long simulation of WT St_βIB1 on a pure POPC bilayer.

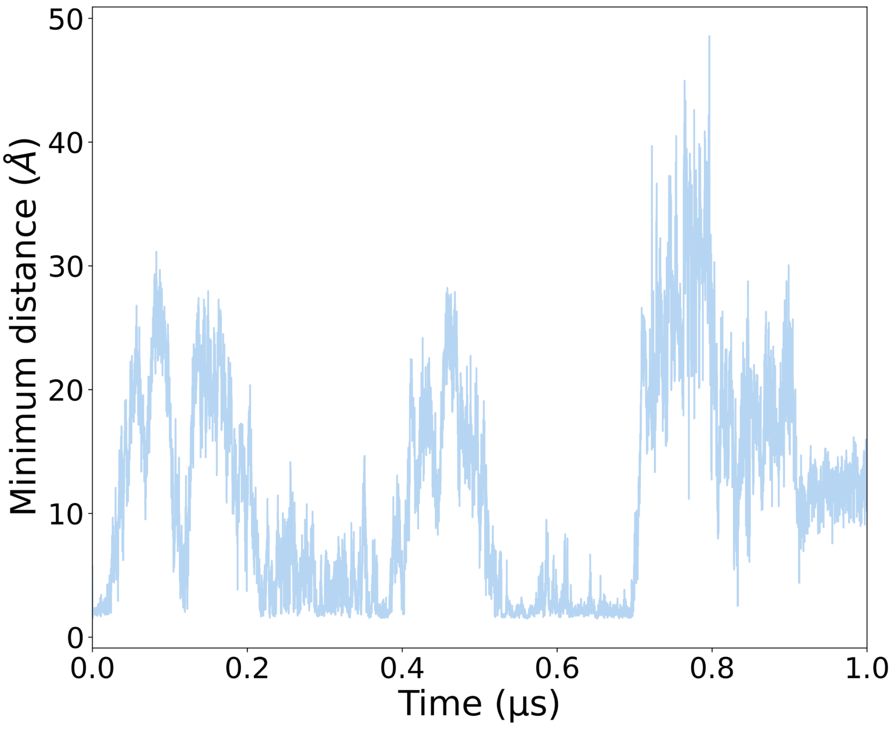

Figure E. Distance between tyrosine 46 and the bilayer in the 1 µs-long simulation of WT St_βIB1 on a pure POPC bilayer. The distance reported here is the distance between the center of mass of Y46 and the average plane of choline nitrogen atoms. The plane is calculated at every frame.

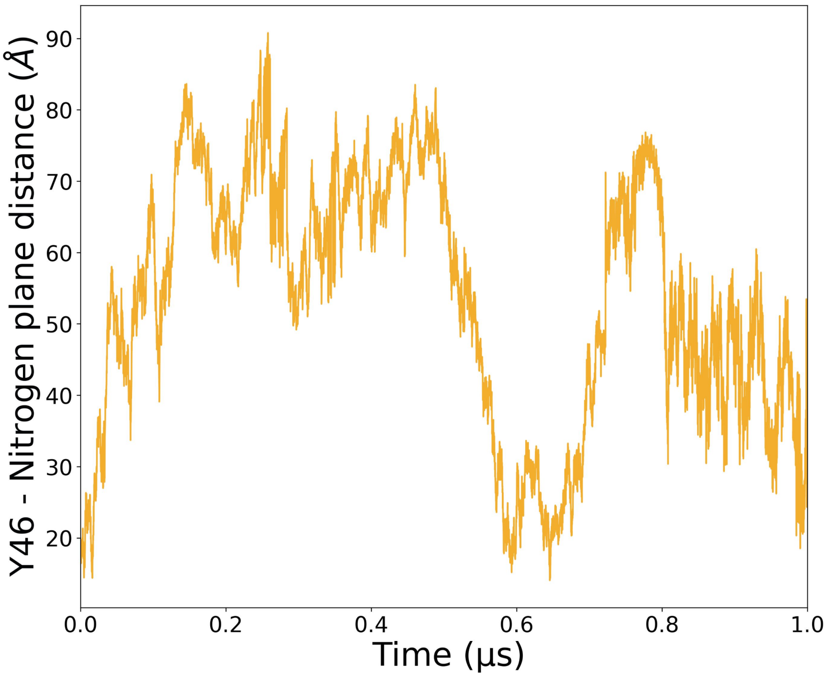

Figure F. Distances between the aromatic cage and the choline group of POPC 23, 63 and 82 during R1 simulation of Li_αIA1 on a pure POPC bilayer. The distance reported here is the distance between the center of mass of the aromatic cycles involved in the aromatic cage and the nitrogen atom of a given lipid (POPC 23, POPC 63 or POPC 82).

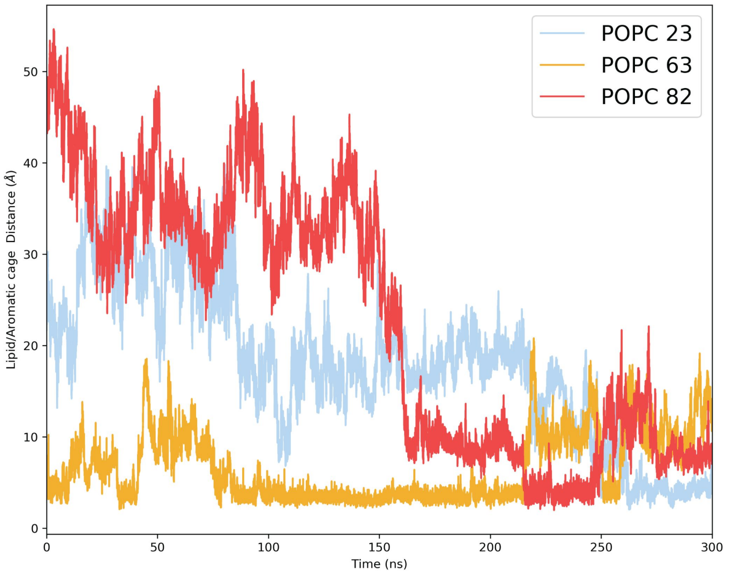

Figure G. Distance between the aromatic cage and the choline group of POPC 12 during R1 simulation of Ll_αIII1 on a pure POPC bilayer. The distance reported here is the distance between the center of mass of the aromatic cycles involved in the aromatic cage and the nitrogen atom of POPC 12.

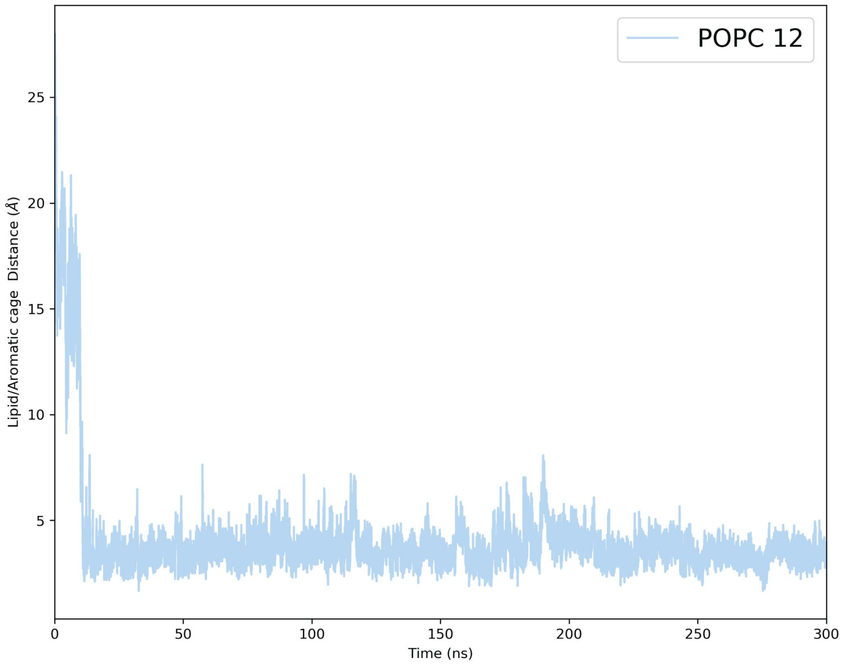

Figure H. Distance between the aromatic cage and the choline group of POPC 124 and 126 during R1 simulation of R44Y/S60Y St_βIB1 on a pure POPC bilayer. The distance reported here is the distance between the center of mass of the aromatic cycles involved in the aromatic cage and the nitrogen atom of POPC 124 or POPC 126.

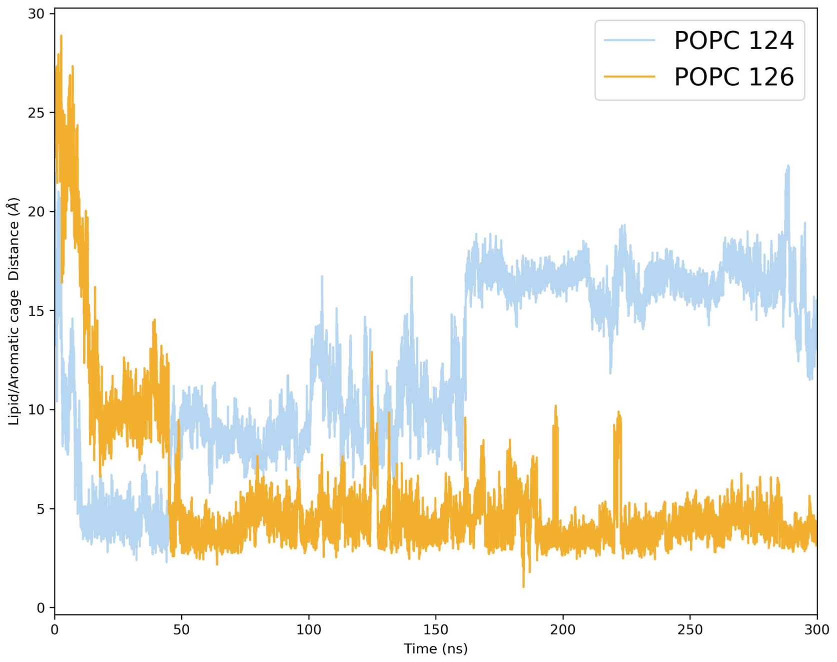

Figure I. Simulation of Li_αIA1 on a POPC:POPE (50:50) bilayer. Distance between the aromatic cage COM and the average plane of the choline nitrogens for replica 1 (A) and replica 2 (B). For replica 2, we also plot the distance between the cage COM and the two lipids observed entering the cage, POPC 35 and POPC 58. The average plane of the choline nitrogens is calculated at every frame. In replica 1, the large distances reflect that the enzyme does not bind to the bilayer, while it does in replica 2, and 2 PC headgroups successively bind to the cage.

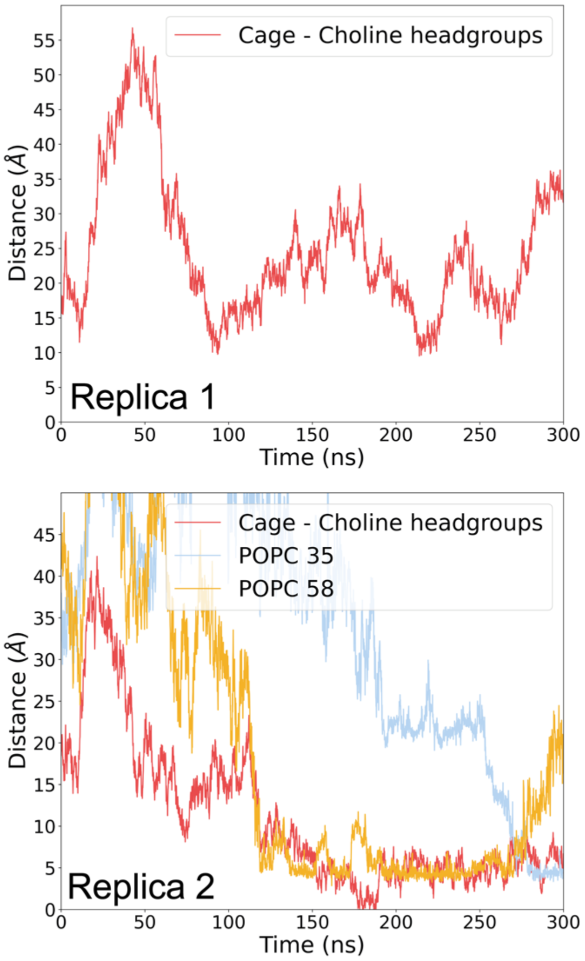

Supplement: S6 Text — (PDF) [file pcbi.1009871.s009.pdf]
