# Supplementary material for: Specificity of Loxosceles α clade phospholipase D enzymes for choline-containing lipids: Role of a conserved aromatic cage
Source: PLoS Comput Biol. 2022 Feb 18;18(2):e1009871. doi: 10.1371/journal.pcbi.1009871 (PMC8893692; doi:10.1371/journal.pcbi.1009871)
Supplement: Attachment — Submitted filename: RevComments_2.pdf [file pcbi.1009871.s012.pdf]

Moutoussamy et al. set out to study a hypothesis that a key reason why a certain set of phospholipases (α-clade) has a higher affinity towards phosphatidylcholine (PC) lipid headgroups than a related set (β-clade), is that α-clade typically contains a structural element dubbed 'aromatic cage' (comprising 2 to 4 tryptophans or tyrosines), whereas β-clade typically lacks such a cage.

They find support for their hypothesis primarily by performing and analysing unbiased all-atom molecular dynamics (MD) simulations; a liposome-binding assay provides additional experimental support.

I find the work reasonable, and aptly executed, and thus likely to be suitable to be published in PLOS Computational Biology. I would, however, like to draw the authors' attention to the following points:

(1) In the Introduction (p5) the authors spell out their hypothesis as:
"[T]he tyrosine and tryptophan residues on the i-face of La_αIB2bi and other α-clade enzymes provides a mechanism to selectively recognize choline-containing lipids as ligands." However, it appears that none of the performed MD simulations with the aromatic-cage containing enzymes had other than PC headgroups in their membranes. Therefore, it is not possible to say if the aromatic cage (composed of the said tyrosine and tryptophan residues on the i-face) recognizes SELECTIVELY the PC headgroups. To test the selective recognition, simulations of membranes containing (also) some other headgroup (such as PE) should be performed. If the stated hypothesis holds true, the α-clade enzymes (or more specifically the aromatic cage in them) would not recognize these other headgroups—or would at least bind them much less frequently than the PC headgroups.

This is an excellent suggestion and we thus performed two simulations (two replicates) with an alpha-clade PLD (Li_αIA1) in the presence of a POPC:POPE (50:50) bilayer. We chose to use a PC:PE (50:50) bilayer, because (i) the ethanolamine ammonium group might also be a candidate for cation-pi interactions with the cage, and (ii) it is the same bilayer as we used for the β-clade enzyme. Using this composition, we showed that the protein binds to the bilayer in only one of the replicas. In the other one, it does bind but more shallowly than to a POPC bilayer. In that replica, the cage specifically binds PC but not PE lipids. We have listed the additional simulations in the Methods section and the results, included new figures (Fig.7, S12, S19) and a table (Table 4), are presented in a new paragraph entitled "(3) Specific recognition of PC headgroups by the α clade enzyme Li_αIA1".

Ok, nice!

(2) The abstract states that: "Here, we confirmed the membrane binding site of α *and β clade PLDs on choline and ethanolamine-containing bilayers, respectively." The statement is acceptable concerning the α-clade—although of course 'confirmed' is a bit strong word for a predominantly MD simulation study—where the binding site (for PC) appears to be the aromatic cage; however, it is not quite clear to me what the confirmed binding site for the β clade on ethanolamine is. Could the authors please clarify?*

With this sentence we meant to convey that we confirmed that the i-face is involved in membrane-binding. This hypothesis was proposed in an earlier publication reporting experimental data (Lajoie et al., J Biol Chem, 2015). We have modified that sentence in the abstract.

Ok. I would, however, not use the abbreviated term "i-face" (alone) in the abstract, but spell it out (also) explicitly as "interfacial face".

***(3) What is the conformation of the PC headgroup bound in the aromatic cage? In** particular, is the conformation something typically seen in the other (free) PC heads? Or does the conformation adapt to the binding site, as recently claimed by the proponents of the so-called 'Inverse Conformational Selection Model' [Bacle et al, JACS 143 13701 (2021), https://doi.org/10.1021/jacs.1c05549]?*

We calculated the angle between the membrane normal and the P-N vector for the POPC lipid interacting with the aromatic cage of Li_αIA1. The result is 50 +/- 20 degrees while the average angle is 63+/-20 degrees for the other POPC lipids. From Figure 2 in Bacle et al. the distribution for the same angle seems to be centered around 65 degrees. Given the breadth of the distribution in Bacle et al, and the standard deviations we observe, we feel that we cannot conclude without providing a significant amount of additional analyses. We would need more analyses not only of our simulations, but also of caged choline groups in experimentally resolved structures to exclude artifacts due to the force field. While this is a very interesting question, we feel that it is a complex one, and not within the scope of this manuscript. We thus suggest not to include that in this work.

Looking at the standard deviations authors give in their reply (20 degrees) and taking into account that there are over 200 PC headgroups in, for example, the simulation of Li_αIA1 in pure POPC, one already sees that the standard error of the mean is 20/sqrt(200)=1.4, that is, less than 2 degrees. Thus for the unbound case the angle is 63 +/- 2 degrees. This suggests that binding to Li_αIA1 indeed does change the conformation of the headgroup, which would be in line with the 'Inverse Conformational Selection Model'.

This suggestion can be further confirmed by including also at the systems 2, 5, 6, and 9 of Table 1. This will allow narrowing the error estimate for the unbound case to below 1 degree, and possibly—if individual lipid-binding events are taken as independent samples—the estimate for the bound case to something like 7 degrees.

I thus do not agree with the authors' reply that it would require a lot of extra work to answer this question, which is, as they say, an interesting and topical one.

***(4) Please make the raw simulation trajectories available on an open data service. A** nice option is using the CERN-run Zenodo (zenodo.org), which is free to use, allows trajectories up to 50 gigabytes, and provides DOIs, which one can then cite directly in the manuscript.*

We uploaded the trajectories to the Norwegian national infrastructure for research data NIRD (https://archive.norstore.no/). The corresponding DOI (10.11582/2021.00099) is given in the Methods section, at the end of the paragraph titled "Molecular dynamics simulations".

Ok.

***(5) When discussing the system preparation, please (i) mention roughly the size (x/** y/z) of the simulation box; (ii) provide the Table S1 in the actual paper; and (iii) include in this table the DOIs for raw MD trajectories, see the previous point.*

The Table S1 has been moved to the main text (as Table 1), and the sizes of the simulation boxes have been added to this table.

Ok.

***(6) It is stated that the two replicas "differ by the equilibration step". What is meant** by this, that is, how do they differ exactly?*

We meant that replicas started from the same minimization step but an independent equilibration was performed for each replica and therefore, different velocity distributions were used for the production run. This is now written in the Methods section in order to make this point clearer.

In the Methods it now reads: “Replicas started from the same minimization step but independent equilibration steps were performed for each replica and therefore, different velocity distributions were used for the production run.”

It is still not explicitly clear to me why the two simulations have different velocities. Were the initial velocities (after minimization) drawn from Maxwell–Boltzmann distribution using different seeds for the random number generator? Or where the same initial velocities used, but a different seed used for the Langevin dynamics, thus leading to an eventual deviation of the two simulation trajectories?

(7) Concerning the ions, was the NB-fix correction of CHARMM36 used?

Yes, the NB-fix correction of CHARMM36 was used. This is now stated in the Methods section.

Ok. Please add also the citation. DOI: 10.1021/jp401512z

(8) What was the saving frequency in the simulations?

Here we are assuming that the reviewer asks about the saving frequency for configurations of the system in trajectory files. This was set to 10ps, and we have added this information to the Methods section.

Ok. Yes, I was after exactly this information, thanks.

***(9) Concerning the pressure control, was it isotropic, that is, all box vectors were** scaled with the same factor?*

The pressure control was semi-isotropic. We controlled the ratio of the unit cell in the x-y plane constant using the useConstantRatio keyword in NAMD. This is now written in the Methods section.

Ok.

(10) Was SHAKE applied also in waters?

SHAKE was applied to water molecules indeed. It is now stated explicitly in the Methods section.

Ok.

***(11) When determining the depth of insertion, the reference location (of the upper** phosphate plane) was "calculated on the last frame of the simulation". Why? To me it seems that this could introduce a systematic error, especially if the membrane shifts vertically during the simulation. To this end, the location of the upper phosphate plane should be determined for each frame separately. In fact, most natural would be to center the trajectory before analysis such that the center of mass of the upper phosphate plane stays at zero, then the z-coordinate can be directly interpreted as the depth of insertion.*

This is absolutely correct. Our original text gave a misleading explanation of how we calculated the depth of anchoring. The depth of anchoring was not calculated along the whole trajectories but only on the last frame of each simulation. The Methods section has been modified to clarify this.

Ok.

***(12) In addition to the protein backbone RMSD shown now in the SI, please show** as a function of simulation time (i) the simulation box area in the bilayer plane, and (ii) the minimum distance between the protein and its nearest image.*

We added the requested analysis on the SI:

- simulation box area along simulation time (Figure S4 to S6)
- minimum distance between the protein and its nearest image along simulation time (Figure S7 to S9)

Ok. Why does in Fig S7 the L1_αIII1 replica 1 data end at 220 ns?

***(13) Please show also the (maximum) depth of insertion as a function simulation** time for all the simulations.*

We would very much like to avoid providing this data. While we have reported this measure before (or at least it average values), we don’t anymore for the following reasons. The distribution of the phosphate groups along z is fairly wide making the average plane of the phosphates a bad reference to measure the variation of the depth of anchorage of individual amino acids. The minimum protein-lipid distances is a good indicator of how stably the proteins are anchored at the bilayer, and we prefer to use this measure.

Ok. However, I am bit confused by authors’ reply that “the average plane of the phosphates [is] a bad reference to measure the variation of the depth of anchorage of individual amino acids.” As far as I understand, it is this measure that is shown in figures 3D, 5D, 7C, and 8D?

(14) On p12 it is written that the "density plots (Fig. 5B) show an anchoring slightly *deeper than for the α-clade enzymes". This is rather hard for the reader to see, so it might be better to give this information as numbers—say, list the time averages of the maximum insertion depths for each enzyme.*

We actually find that comparing Figures 5B and 5D to Figures 3A (top panel) and 3D, respectively, actually shows a clear difference between the β and α clade enzymes in terms of their depth of insertion. We have rephrased the paragraph on page 12 to make this clearer: "The density plots (Fig. 5B) show an anchoring slightly deeper than for the α-clade enzymes in the PC-containing bilayers (Fig.3A, top panel). This is also visible from the insertion at the end of the simulation which is deeper for the catalytic loop (β2α2) and the flexible loop (β6α6) of St_βIB1 (Fig. 5D and Table S4) than for the corresponding loops in the α-clade enzymes (Fig.3D and Table S2)."

Regarding time averages of insertion depths along simulation time, we refer the reviewer to our answer to comment (13) above.

Ok.

(15) In Fig. 7D, please rotate the color bar showing the insertion depth, such that it *intuitively matches the snapshot (negative numbers bottom, positive top).*

This is an excellent suggestion. We have updated Figures 3, 5, 7 (new) and 8 according to this comment.

Ok.

(16) In Fig 8, the top panel says "Lar_aIIB2bi", but the caption "La_αIB2bi".

We thank the reviewer for pointing this mistake. The right name is La_αIB2bi. The label on the figure has been corrected.

Ok.

(17) In Fig 8, what do the percentages stand for? Is it correct that for each experiment *the "S" and "P" percentages (out of which only the "P" is shown), will add to 100? If yes, then I think it would be easier for the reader, if also the "S" percentages were written on the plot.*

Yes, it is correct. We updated the figure (now Fig.9) with the percentage related to the supernatant (S).

Ok.

***(18) When discussing the Fig. 8 in the text, two numbers are given: 15+/-4% and** 28+/-4%. How were these exactly calculated?*

Peak areas for the bands in each gel lane were measured using ImageJ. The percent of the protein bound by the liposomes was calculated from the area of the band in the pellet lane divided by the total area of the pellet and supernatant bands. This percent was then corrected to subtract the apparent percent bound observed in a negative control (run on the same gel) in which no liposome was added to a sample of the same protein. The measured percent binding from three independent runs (including different protein preps) were averaged and the uncertainty reported as the standard error of the mean. This is how we arrived at 15 ± 4% binding for wt and 28 ± 4% binding for the mutant. We also note that as stated in the Figure 9 caption, the percent bound was always higher for the mutant (by 10-17%) in each of the three independent measurements, giving an increase in binding of 13 ± 2% using standard error of the mean.

What I still find confusing is that these numbers (15% and 28%) do not appear in Fig 9. If I now understand correctly, the number 23% in Fig 9 is the value for this particular experiment, and the corresponding value 15 ± 4% in the text is the value from all three repeats? And again 36% is for this particular experiment, and 28 ± 4% for all the three repeats? Maybe all the numbers for the three independent runs could be given in a table, so the reader could work out on their own, how these different numbers are connected?

***(19) Accuracies in tables. The H-bond and cation-pi occupancies are not significant** to tenths of promilles; I would expect to see them maximally in the precision of percentages. Same for hydrophobic contacts; probably a hundreth of a contact (as in Table 2) is not really significant?*

We have modified Tables 2 to 5 accordingly, with average number of contacts and percentages for hydrogen bond occupancies now rounded to one decimal place. We have also modified the number of decimal places for anchoring depths in Tables S2 to S4.

Ok.

(20) What is the cyan ball shown in the snapshots of Fig 4? An ion?

The cyan ball is a magnesium ion located in the catalytic site. We have updated the legend of Figure 4, and added a sentence in the Methods section (System setup) to inform about the presence of the Mg++ ion in the X-ray structures.

Ok.

(21) Typos:

Corrections ok. Just a couple new ones that caught my eye:

Across the manuscript, both ‘L1_αIII1i’ and ‘L1_αIII1’ are used. Maybe the ending ‘i’s could be consistently removed?

Also, the characters ‘_’ and ‘-’ in protein names are being both used, i.e., there is for example both ‘Li_αIA1’ and ‘Li-αIA1’ as well as ‘L1_αIII1’ and ‘L1-αIII1’.

p6: ‘Clustal Omega (35) was used to align multiple sequences ~~alignment~~.’

p8: ‘The composition of the ~~eight~~ **nine** simulated systems.’

p9: ‘the distance between the hydrogen and the ~~donor~~ **acceptor** of hydrogen bond atom should be below or equal to 2.4 Å’

p15: ‘POPC:POPE bilayer. The hydrogen **bond** network between Li_αIA1 and the bilayer’

p19: Missing citation: ’This result is consistent with the observation made by Cheng et al. **(27)** Indeed, when’

Caption of Figure S12: ‘Minimum protein-bilayer distance in simulations of Li_αIA1~~,~~ ~~Ll_αIII1,~~ and WT St_βIB1 ~~and R44Y/S60Y~~ with a POPC:POPE (50:50) bilayer. Replica 1 is represented in orange and replica 2 in cyan.’
