# Supplementary material for: Specificity of Loxosceles α clade phospholipase D enzymes for choline-containing lipids: Role of a conserved aromatic cage
Source: PLoS Comput Biol. 2022 Feb 18;18(2):e1009871. doi: 10.1371/journal.pcbi.1009871 (PMC8893692; doi:10.1371/journal.pcbi.1009871)
Supplement: Attachment — Submitted filename: Review of PCOMPBIOL.docx [file pcbi.1009871.s010.docx]

**Review of PCOMPBIOL-D-21-01515**

This paper uses MD simulations to analyse the specificity for lipids of phospholipases D from spider toxins. In particular, this study is concerned with the role of a conserved aromatic cage in providing specificity for choline headgroups of lipids such as PC and sphingomyelin. The simulations support the key role of the aromatic cage in choline recognition. These results are supported by in vitro liposome binding assays.

This is an interesting and important study of membrane lipid recognition. It is also of more general interest given the importance of aromatic/choline interactions in a number of receptors e.g. the acetylcholine receptor. (Infield et al. (2021) https://doi.org/10.1016/j.jmb.2021.167035 provide a nice recent review of this.) Perhaps this could be mentioned in the current manuscript. The simulations have been performed and analysed carefully, and the data support the conclusions drawn. I have just a couple of suggestions/comments which the authors may like to address.

1. Membranes explored include: POPC and POPC/SM/Chol. I wonder what would happen with the alpha-clade toxins with a PC-free membrane as a control. For example, one could use a PE/PG bilayer, which has been employed in simulation studies as a simple model of a bacterial membrane.
2. As discussed in detail in some of Reuter’s previous papers (e.g. ref 25) there is some debate as to how well additive forcefields such as CHARMM can capture cation-pi interactions. This caveat should be mentioned in the current paper, perhaps with a reference to a recent paper (Rupakheti et al. (2018) https://doi.org/10.1007/s00214-018-2376-z) which compares additive (Charmm) and polarisable (Charmm/Drude) forcefields for model cation-pi complexes.
