# Supplementary material for: Specificity of Loxosceles α clade phospholipase D enzymes for choline-containing lipids: Role of a conserved aromatic cage
Source: PLoS Comput Biol. 2022 Feb 18;18(2):e1009871. doi: 10.1371/journal.pcbi.1009871 (PMC8893692; doi:10.1371/journal.pcbi.1009871)
Supplement: Attachment — Submitted filename: MoutoussamyE_Response_to_Reviewers_R1.docx [file pcbi.1009871.s011.docx]

We are thankful to the three reviewers for their very valuable comments. Our responses to all comments and questions are provided below and changes to the manuscript are highlighted with red fonts in the main text.

**Reviewer 1**

*This manuscript presents mainly a set of molecular dynamics (MD) simulations of spider venom PLD structures focusing on the aromatic cage and interactions with the PC headgroup. Comparisons are made with a protein in the b clade (St_bIB1) and how it interacts with the membrane when lacking this aromatic cage. Overall, this work is interesting but there lacks proof of equilibration of membrane protein interaction and statistics associated with the experimental work. Details on this and other aspects are provided below.*

*General Issues:*

*1. Membrane-protein equilibration: The authors focus on a simple metric of RMSD of protein structure but do not focus on presenting a key metric associated with the*

*proteins binding to the membrane. There needs to be some quantitative proof that the proposed bound states are near equilibrium. Key plots of distance of the protein relative to a metric in the membrane is a start. Moreover, plots of block averages of protein density profiles in comparison with the membrane is important. The concern is that the binding of peripheral membrane proteins can be more on the microsecond timescale and either long simulations or enhanced sampling is typically needed to probe stable binding.*

We are aware of examples of binding of peripheral membrane proteins on the microsecond timescale, but it can also be much faster (Cf <https://doi.org/10.1016/j.jmb.2012.08.015>, <https://doi.org/10.1016/j.jmb.2013.05.026>) owing to the diversity of peripheral proteins.

We nevertheless agree very much with the reviewer that it would be useful for the reader to see minimum distance plots and have added those as Supporting Information for each simulation (Figures S9 to S13). We would rather not add plots of block average protein density as they would not bring any useful additional information, but would result in adding a large number of figures to the SI.

*2. St_bIB1 with POPC: The authors base low affinity on how this protein doesn’t bind to the POPC membranes but experimental with SM/Chol membrane suggests some binding. It appears that the timescale might not be enough (300ns) to probe binding for these PC headgroups. Have you tested this binding with longer simulations or with enhanced sampling?*

We extended to 1 microsecond one of the two simulations of the beta clade PLD St_βIB1 (PDBid: 3RLH) with a POPC bilayer. As illustrated by the minimum protein-bilayer distance along simulation time (Fig. S13) the enzyme approaches the bilayer on several occasion but did not find a stable orientation, as illustrated by the large range of variations of the distance between Y46 of the catalytic loop and the bilayer (Fig S14).

*3. Liposome Binding Assay: The binding assay is relatively crude and the figure lacks statistical information and the text on page 8 suggests some variance but unclear how this was obtain or if this is standard error or standard deviation. Quantifying intensity of bands with numerical values is qualitative at best with this method. Can one statistically state that the mutated St_bIB1 shows more binding? The band intensity suggest maybe but is this reproducible and do you have the same amount of mutated protein in comparison with the wildtype?*

Yes, statistically we can state that the Y44/Y60 variant shows more binding, based on standard errors of the mean of three determinations. The effect is not huge, but it is reproducible, and the amounts of the wild-type and mutant used are equivalent.

*4. St_bIB1 other factors in binding: The liposomal assay suggests other factors are*

*contributing to weak binding beyond the lack of an aromatic cage. The discussion lacks potential details on what the other factors might be. Can simulations provide some insight?*

This is an interesting question and an active area of investigation. To date we have not identified mutations (other than the aromatic cage substitutions) that further increase the binding of St_βIΒ1 either in simulation or in experiment, though this is an active ongoing exploration. In addition, we do not see obvious indications from the simulations of alpha-clade members binding to choline-rich bilayers of what other key interactions might be missing in St_βIΒ1. Thus, at this point, we don’t feel justified to speculate what the other factors could be.

*Specific Comments*

*(1) Abstract: Please define PLD.*

This is now corrected.

*(2) Ionizable residues: Do you have ionizable residue with this protein? Was a pKa estimate made on the protein to verify the proper states of these residues?*

Thank you for noting this. Ιndeed, we evaluated the protonation state of ionizable side chains using PROPKA3 and none of the amino acids had a pKa that deviated significantly from the standard values for the isolated residues. We have updated the Methods section to reflect this.

*(3) Initial protein setup: The authors provide a center of mass distance describing the placement of the protein relative to the center of the bilayer. This is not that informative and should be supplemented with a measure of minimal distance with the protein or some other distance related to key membrane binding domain/s on the protein.*

We thank the reviewer for this suggestion. The minimum protein-bilayer distance (4-6 Å) is now reported in the Methods section.

*Figure 3A: The density profiles are not described in the figure or figure captions. I can figure this out but the protein vs. membrane density should be clearly stated.*

*Cutoff LJ: The authors forgot to mention the cutoff method and length used in these*

*simulations.*

We thank the reviewer for pointing at this and apologize for the uncomplete legend of Figure 3. We corrected that legend in the revised version of the manuscript. We also added information about the LJ cutoff in the Methods section.

**Reviewer 2**

*This paper uses MD simulations to analyse the specificity for lipids of phospholipases D from spider toxins. In particular, this study is concerned with the role of a conserved aromatic cage in providing specificity for choline headgroups of lipids such as PC and sphingomyelin. The simulations support the key role of the aromatic cage in choline recognition. These results are supported by in vitro liposome binding assays.*

*This is an interesting and important study of membrane lipid recognition. It is also of more general interest given the importance of aromatic/choline interactions in a number of receptors e.g. the acetylcholine receptor. (Infield et al. (2021) https://doi.org/10.1016/j.jmb.2021.167035 provide a nice recent review of this.) Perhaps this could be mentioned in the current manuscript. The simulations have been performed and analysed carefully, and the data support the conclusions drawn. I have just a couple of suggestions/comments which the authors may like to address.*

1. *Membranes explored include: POPC and POPC/SM/Chol. I wonder what would happen with the alpha-clade toxins with a PC-free membrane as a control. For example, one could use a PE/PG bilayer, which has been employed in simulation studies as a simple model of a bacterial membrane.*

This is an excellent suggestion and related to that of Reviewer 3, who suggested to test the selective recognition of PC on bilayers containing also other lipids. We chose to simulate Li_αIA1 on a PC:PE (50:50) bilayer, because (i) the ethanolamine ammonium group might also be a candidate for cation-pi interactions with the cage, and (ii) it is the same bilayer as we used for the β-clade enzyme. Using this composition, we showed that the protein binds stably to the bilayer in only one of the replicas (R2). It binds more shallowly than to a POPC bilayer (Cf Fig.7). In that replica the cage specifically binds PC lipids, PE lipids are never observed in the cage. We have listed the additional simulations in the Methods section and the results, including new figures (Fig.7, S12, S19) and a table (Table 4), are presented in a new paragraph entitled “(3) Specific recognition of PC headgroups by the α clade enzyme Li_αIA1”.

Also, we felt that a PC:PG bilayer was not a very relevant model for the membrane targeted by the *SicTox* PLDs.

2. *As discussed in detail in some of Reuter’s previous papers (e.g. ref 25) there is some debate as to how well additive forcefields such as CHARMM can capture cation-pi interactions. This caveat should be mentioned in the current paper, perhaps with a reference to a recent paper (Rupakheti et al. (2018) https://doi.org/10.1007/s00214-018-2376-z) which compares additive (Charmm) and polarisable (Charmm/Drude) forcefields for model cation-pi complexes.*

We thank the reviewer for this suggestion and have inserted a reference to this paper in the Methods section.

**Reviewer 3**

*Moutoussamy et al. set out to study a hypothesis that a key reason why a certain set of phospholipases (α-clade) has a higher affinity towards phosphatidylcholine (PC) lipid headgroups than a related set (β-clade), is that α-clade typically contains a structural element dubbed 'aromatic cage' (comprising 2 to 4 tryptophans or tyrosines), whereas β-clade typically lacks such a cage.*

*They find support for their hypothesis primarily by performing and analysing unbiased all-atom molecular dynamics (MD) simulations; a liposome-binding assay provides additional experimental support.*

*I find the work reasonable, and aptly executed, and thus likely to be suitable to be published in PLOS Computational Biology. I would, however, like to draw the authors' attention to the following points:*

*(1) In the Introduction (p5) the authors spell out their hypothesis as:*

*"[T]he tyrosine and tryptophan residues on the i-face of La_αIB2bi and other α-clade enzymes provides a mechanism to selectively recognize choline-containing lipids as ligands." However, it appears that none of the performed MD simulations with the aromatic-cage containing enzymes had other than PC headgroups in their membranes. Therefore, it is not possible to say if the aromatic cage (composed of the said tyrosine and tryptophan residues on the i-face) recognizes SELECTIVELY the PC headgroups. To test the selective recognition, simulations of membranes containing (also) some other headgroup (such as PE) should be performed. If the stated hypothesis holds true, the α-clade enzymes (or more specifically the aromatic cage in them) would not recognize these other headgroups—or would at least bind them much less frequently than the PC headgroups.*

This is an excellent suggestion and we thus performed two simulations (two replicates) with an alpha-clade PLD (Li_αIA1) in the presence of a POPC:POPE (50:50) bilayer. We chose to use a PC:PE (50:50) bilayer, because (i) the ethanolamine ammonium group might also be a candidate for cation-pi interactions with the cage, and (ii) it is the same bilayer as we used for the β-clade enzyme. Using this composition, we showed that the protein binds to the bilayer in only one of the replicas. In the other one, it does bind but more shallowly than to a POPC bilayer. In that replica, the cage specifically binds PC but not PE lipids. We have listed the additional simulations in the Methods section and the results, included new figures (Fig.7, S12, S19) and a table (Table 4), are presented in a new paragraph entitled “(3) Specific recognition of PC headgroups by the α clade enzyme Li_αIA1”.

*(2) The abstract states that: "Here, we confirmed the membrane binding site of α and β clade PLDs on choline and ethanolamine-containing bilayers, respectively." The statement is acceptable concerning the α-clade—although of course 'confirmed' is a bit strong word for a predominantly MD simulation study—where the binding site (for PC) appears to be the aromatic cage; however, it is not quite clear to me what the confirmed binding site for the β clade on ethanolamine is. Could the authors please clarify?*

With this sentence we meant to convey that we confirmed that the i-face is involved in membrane-binding. This hypothesis was proposed in an earlier publication reporting experimental data (Lajoie et al., J Biol Chem, 2015). We have modified that sentence in the abstract.

*(3) What is the conformation of the PC headgroup bound in the aromatic cage? In particular, is the conformation something typically seen in the other (free) PC heads? Or does the conformation adapt to the binding site, as recently claimed by the proponents of the so-called 'Inverse Conformational Selection Model' [Bacle et al, JACS 143 13701 (2021),* [*https://doi.org/10.1021/jacs.1c05549*](https://doi.org/10.1021/jacs.1c05549)*]?*

We calculated the angle between the membrane normal and the P-N vector for the POPC lipid interacting with the aromatic cage of Li_αIA1. The result is 50 +/- 20 degrees while the average angle is 63+/-20 degrees for the other POPC lipids. From Figure 2 in *Bacle et al.* the distribution for the same angle seems to be centered around 65 degrees. Given the breadth of the distribution in *Bacle et al,* and the standard deviations we observe, we feel that we cannot conclude without providing a significant amount of additional analyses. We would need more analyses not only of our simulations, but also of caged choline groups in experimentally resolved structures to exclude artifacts due to the force field. While this is a very interesting question, we feel that it is a complex one, and not within the scope of this manuscript. We thus suggest not to include that in this work.

*(4) Please make the raw simulation trajectories available on an open data service. A nice*

*option is using the CERN-run Zenodo (zenodo.org), which is free to use, allows*

*trajectories up to 50 gigabytes, and provides DOIs, which one can then cite directly in*

*the manuscript.*

We uploaded the trajectories to the Norwegian national infrastructure for research data NIRD (https://archive.norstore.no/). The corresponding DOI (10.11582/2021.00099) is given in the Methods section, at the end of the paragraph titled “*Molecular dynamics simulations*”.

*(5) When discussing the system preparation, please (i) mention roughly the size (x/y/z) of the simulation box; (ii) provide the Table S1 in the actual paper; and (iii) include in this table the DOIs for raw MD trajectories, see the previous point.*

The Table S1 has been moved to the main text (as Table 1), and the sizes of the simulation boxes have been added to this table.

*(6) It is stated that the two replicas "differ by the equilibration step". What is meant by this, that is, how do they differ exactly?*

We meant that replicas started from the same minimization step but an independent equilibration was performed for each replica and therefore, different velocity distributions were used for the production run. This is now written in the Methods section in order to make this point clearer.

*(7) Concerning the ions, was the NB-fix correction of CHARMM36 used?*

Yes, the NB-fix correction of CHARMM36 was used. This is now stated in the Methods section.

*(8) What was the saving frequency in the simulations?*

Here we are assuming that the reviewer asks about the saving frequency for configurations of the system in trajectory files. This was set to 10ps, and we have added this information to the Methods section.

*(9) Concerning the pressure control, was it isotropic, that is, all box vectors were scaled*

*with the same factor?*

The pressure control was semi-isotropic. We controlled the ratio of the unit cell in the x-y plane constant using the useConstantRatio keyword in NAMD. This is now written in the Methods section.

*(10) Was SHAKE applied also in waters?*

SHAKE was applied to water molecules indeed. It is now stated explicitly in the Methods section.

*(11) When determining the depth of insertion, the reference location (of the the upper phosphate plane) was "calculated on the last frame of the simulation". Why? To me it seems that this could introduce a systematic error, especially if the membrane shifts vertically during the simulation. To this end, the location of the upper phosphate plane should be determined for each frame separately. In fact, most natural would be to center the trajetory before analysis such that the center of mass of the upper phosphate plane stays at zero, then the z-coordinate can be directly interpreted as the depth of insertion.*

This is absolutely correct. Our original text gave a misleading explanation of how we calculated the depth of anchoring. The depth of anchoring was not calculated along the whole trajectories but only on the last frame of each simulation. The Methods section has been modified to clarify this.

*(12) In addition to the protein backbone RMSD shown now in the SI, please show as a function of simulation time (i) the simulation box area in the bilayer plane, and (ii) the minimum distance between the protein and its nearest image.*

We added the requested analysis on the SI:

- simulation box area along simulation time (Figure S4 to S6)
- minimum distance between the protein and its nearest image along simulation time (Figure S7 to S9)

*(13) Please show also the (maximum) depth of insertion as a function simulation time for all the simulations.*

We would very much like to avoid providing this data. While we have reported this measure before (or at least it average values), we don’t anymore for the following reasons. The distribution of the phosphate groups along z is fairly wide making the average plane of the phosphates a bad reference to measure the variation of the depth of anchorage of individual amino acids. The minimum protein-lipid distances is a good indicator of how stably the proteins are anchored at the bilayer, and we prefer to use this measure.

*(14) On p12 it is written that the "density plots (Fig. 5B) show an anchoring slightly deeper than for the α-clade enzymes". This is rather hard for the reader to see, so it might be better to give this information as numbers—say, list the time averages of the maximum insertion depths for each enzyme.*

We actually find that comparing Figures 5B and 5D to Figures 3A (top panel) and 3D, respectively, actually shows a clear difference between the β and α clade enzymes in terms of their depth of insertion. We have rephrased the paragraph on page 12 to make this clearer: “The density plots (Fig. 5B) show an anchoring slightly deeper than for the α-clade enzymes in the PC-containing bilayers (Fig.3A, top panel). This is also visible from the insertion at the end of the simulation which is deeper for the catalytic loop (β2α2) and the flexible loop (β6α6) of St_βIB1 (Fig. 5D and Table S4) than for the corresponding loops in the α-clade enzymes (Fig.3D and Table S2).”

Regarding time averages of insertion depths along simulation time, we refer the reviewer to our answer to comment (13) above.

*(15) In Fig. 7D, please rotate the color bar showing the insertion depth, such that it intuitively matches the snapshot (negative numbers bottom, positive top).*

This is an excellent suggestion. We have updated Figures 3, 5, 7 (new) and 8 according to this comment.

*(16) In Fig 8, the top panel says "Lar_aIIB2bi", but the caption "La_αIB2bi".*

We thank the reviewer for pointing this mistake. The right name is La_αIB2bi. The label on the figure has been corrected.

*(17) In Fig 8, what do the percentages stand for? Is it correct that for each experiment the "S" and "P" percentages (out of which only the "P" is shown), will add to 100? If yes, then I think it would be easier for the reader, if also the "S" percentages were written on the plot.*

Yes, it is correct. We updated the figure (now Fig.9) with the percentage related to the supernatant (S).

*(18) When discussing the Fig. 8 in the text, two numbers are given: 15+/-4% and 28+/-4%. How were these exactly calculated?*

Peak areas for the bands in each gel lane were measured using ImageJ. The percent of the protein bound by the liposomes was calculated from the area of the band in the pellet lane divided by the total area of the pellet and supernatant bands. This percent was then corrected to subtract the apparent percent bound observed in a negative control (run on the same gel) in which no liposome was added to a sample of the same protein. The measured percent binding from three independent runs (including different protein preps) were averaged and the uncertainty reported as the standard error of the mean. This is how we arrived at 15 ± 4% binding for wt and 28 ± 4% binding for the mutant. We also note that as stated in the Figure 9 caption, the percent bound was always higher for the mutant (by 10-17%) in each of the three independent measurements, giving an increase in binding of 13 ± 2% using standard error of the mean.

*(19) Accuracies in tables. The H-bond and cation-pi occupancies are not significant to tenths of promilles; I would expect to see them maximally in the precision of percentages. Same for hydrophobic contacts; probably a hundreth of a contact (as in Table 2) is not really significant?*

We have modified Tables 2 to 5 accordingly, with average number of contacts and percentages for hydrogen bond occupancies now rounded to one decimal place. We have also modified the number of decimal places for anchoring depths in Tables S2 to S4.

*(20) What is the cyan ball shown in the snapshots of Fig 4? An ion?*

The cyan ball is a magnesium ion located in the catalytic site. We have updated the legend of Figure 4, and added a sentence in the Methods section (*System setup*) to inform about the presence of the Mg^++^ ion in the X-ray structures.

*(21) Typos:*

*- p3, 3rd last line: differences in is the active -> differences in the active*

Corrected

*- p4, 7th last line: L1_αIII1 has two tyrosines (Y44, Y46, Y62) -> L1_αIII1 has three tyrosines (Y44, Y46, Y62)*

Corrected

*- p7, 5th line: The protein were -> The proteins were*

Corrected

*- p8, 6th last line: 10ps -> 10 ps*

Corrected

*- p10, 10th last line: diffuses away rapidly (Fig. 2 and 3A) -> diffuses away (Fig. 2 and 3A) [The speed of diffusion can not really be reliably determined from two simulations, I would expect?]*

Corrected

*- p10, 8th last line: Figure 4A -> Figure 3A*

Corrected

*- p10, 7th last line: (EDP) and the depth of insertion below -> (EDP) and Fig. 3D the depth of insertion of Li_αIA1 below*

Corrected

*- p10, 6th last line: The two enzymes -> The two α-clade enzymes*

Corrected

*- p10, 2nd last line: with the lipids chains -> with the lipid chains*

Corrected

- p11, 9th last line: 27.54 -> 28

Corrected

- p12, 3rd line: lipids. - The binding -> lipids. The binding

Corrected

- p12, 4th line: molecular docking and -> molecular docking [CITATION] and

Corrected, the reference was moved from the end of the sentence to after “molecular docking”.

- p13, last line: Fig. 8 -> Fig. 7A

Corrected

- p16, 10th last line: ammonium which positive -> ammonium whose positive

Corrected

- p18, 9th last line: MSA indicate that the-terminus -> MSA indicate that the [N?/C?]-terminus

Corrected (C-terminus)

- caption of Table 1: Asterix -> Asterisk

Corrected
