# Supplementary material for: Specificity of Loxosceles α clade phospholipase D enzymes for choline-containing lipids: Role of a conserved aromatic cage
Source: PLoS Comput Biol. 2022 Feb 18;18(2):e1009871. doi: 10.1371/journal.pcbi.1009871 (PMC8893692; doi:10.1371/journal.pcbi.1009871)
Supplement: Attachment — Submitted filename: MoutoussamyE_Response_to_Reviewers_R2.docx [file pcbi.1009871.s013.docx]

We are thankful to the reviewer for their careful read of our manuscript. Our responses to their comments and questions are provided below (see labels Authors – R2), and changes to the manuscript are highlighted with red fonts in the main text.

**Reviewer 3**

*(2) The abstract states that: "Here, we confirmed the membrane binding site of α and β clade PLDs on choline and ethanolamine-containing bilayers, respectively." The statement is acceptable concerning the α-clade—although of course 'confirmed' is a bit strong word for a predominantly MD simulation study—where the binding site (for PC) appears to be the aromatic cage; however, it is not quite clear to me what the confirmed binding site for the β clade on ethanolamine is. Could the authors please clarify?*

**Authors - R1:** With this sentence we meant to convey that we confirmed that the i-face is involved in membrane-binding. This hypothesis was proposed in an earlier publication reporting experimental data (Lajoie et al., J Biol Chem, 2015). We have modified that sentence in the abstract.

***Reviewer R2:*** *Ok. I would, however, not use the abbreviated term “i-face” (alone) in the abstract, but spell it out (also) explicitly as “interfacial face”*

**Authors - R2:** The term “i-face” refers to a particular region of the protein, distinct from the catalytic site, and has been used extensively as such in the literature for many years. It is not an abbreviation of *interfacial* face, although, as we explain in the introduction, the “i” obviously historically originates from the word *interfacial*. We would then rather use “i-face” in the abstract so that readers working in the field of phospholipases will more readily understand which region of the enzymes we are referring to.

*(3) What is the conformation of the PC headgroup bound in the aromatic cage? In particular, is the conformation something typically seen in the other (free) PC heads? Or does the conformation adapt to the binding site, as recently claimed by the proponents of the so-called 'Inverse Conformational Selection Model' [Bacle et al, JACS 143 13701 (2021),* [*https://doi.org/10.1021/jacs.1c05549*](https://doi.org/10.1021/jacs.1c05549)*]?*

**Authors - R1:** We calculated the angle between the membrane normal and the P-N vector for the POPC lipid interacting with the aromatic cage of Li_αIA1. The result is 50 +/- 20 degrees while the average angle is 63+/-20 degrees for the other POPC lipids. From Figure 2 in *Bacle et al.* the distribution for the same angle seems to be centered around 65 degrees. Given the breadth of the distribution in *Bacle et al,* and the standard deviations we observe, we feel that we cannot conclude without providing a significant amount of additional analyses. We would need more analyses not only of our simulations, but also of caged choline groups in experimentally resolved structures to exclude artifacts due to the force field. While this is a very interesting question, we feel that it is a complex one, and not within the scope of this manuscript. We thus suggest not to include that in this work.

*Reviewer R2: Looking at the standard deviations authors give in their reply (20 degrees) and taking into account that there are over 200 PC headgroups in, for example, the simulation of Li_αIA1 in pure POPC, one already sees that the standard error of the mean is 20/sqrt(200)=1.4, that is, less than 2 degrees. Thus for the unbound case the angle is 63 +/- 2 degrees. This suggests that binding to Li_αIA1 indeed does change the conformation of the headgroup, which would be in line with the 'Inverse Conformational Selection Model’.*

*This suggestion can be further confirmed by including also at the systems 2, 5, 6, and 9 of Table 1. This will allow narrowing the error estimate for the unbound case to below 1 degree, and possibly—if individual lipid-binding events are taken as independent samples— the estimate for the bound case to something like 7 degrees.*

*I thus do not agree with the authors’ reply that it would require a lot of extra work to answer this question, which is, as they say, an interesting and topical one.*

**Authors - R2:** We fear that the reviewer misunderstood our answer; the reason for not providing the requested analysis was not because we thought it would require too much work analysing our simulations, but because of (1) we do not see significant differences and (2) we cannot confidently conclude because of the uncertainties related to the ability of the force field to reproduce the correct geometry of caged PC lipids. The latter would require extensive work to verify, including analysis of structures deposited in the PDB and possibly quantum chemistry reference calculations (as we have done for complexes formed of a choline and a single aromatic group, Cf Refs 50-51). This we felt was not within the scope of the presented work.

To comply with the reviewer’s request we have now added the analysis of the POPC headgroup orientations for bound and unbound lipids, for systems 1, 2, 5, 6 and 9 (Table S6). We do not find statistically significant differences between bound and unbound lipids, even when considering standard errors of the mean and treating individual lipid-binding events as independent samples: the averages ± SEM are in 69.7±1.0 degrees for the unbound lipids and 61.4±10.0 for the bound lipids. That being said, we cannot rule out the *inverse conformational selection model* of *Bacle et al*, in the absence of a thorough evaluation of the force field. This is written in the Discussion section.

*(6) It is stated that the two replicas "differ by the equilibration step". What is meant by this, that is, how do they differ exactly?*

**Authors - R1:** We meant that replicas started from the same minimization step but an independent equilibration was performed for each replica and therefore, different velocity distributions were used for the production run. This is now written in the Methods section to make this point clearer.

**Reviewer – R2:** It is still not explicitly clear to me why the two simulations have different velocities. Were the initial velocities (after minimization) drawn from Maxwell–Boltzmann distribution using different seeds for the random number generator? Or where the same initial velocities used, but a different seed used for the Langevin dynamics, thus leading to an eventual deviation of the two simulation trajectories?

**Authors – R2:** The former is correct. We see the need for further clarifying this in the text. We have now changed the above-mentioned sentence to: “*Replicas started from the same minimized structure. Independent equilibration steps were performed for each replica using different velocity distributions. The velocity distributions were generated using different seeds for the random number generator. The equilibration run of each replica was followed by a production run (see above for details on production runs).*”

*(7) Concerning the ions, was the NB-fix correction of CHARMM36 used?*

**Authors - R1:** Yes, the NB-fix correction of CHARMM36 was used. This is now stated in the Methods section.

***Reviewer – R2:*** *Ok. Please add also the citation. DOI: 10.1021/jp401512z*

**Authors – R2:** The reference has been added.

*(12) In addition to the protein backbone RMSD shown now in the SI, please show as a function of simulation time (i) the simulation box area in the bilayer plane, and (ii) the minimum distance between the protein and its nearest image.*

**Authors - R1:** We added the requested analysis on the SI:

- simulation box area along simulation time (Figure S4 to S6)
- minimum distance between the protein and its nearest image along simulation time (Figure S7 to S9)

***Reviewer – R2:*** *Ok. Why does in Fig S7 the L1_αIII1 replica 1 data end at 220 ns?*

**Authors – R2:** This is an unfortunate mistake, we have added the correct plot in Fig S7.

*(13) Please show also the (maximum) depth of insertion as a function simulation time for all the simulations.*

**Authors - R1:** We would very much like to avoid providing this data. While we have reported this measure before (or at least it average values), we don’t anymore for the following reasons. The distribution of the phosphate groups along z is fairly wide making the average plane of the phosphates a bad reference to measure the variation of the depth of anchorage of individual amino acids. The minimum protein-lipid distances is a good indicator of how stably the proteins are anchored at the bilayer, and we prefer to use this measure.

***Reviewer – R2:*** *Ok. However, I am bit confused by authors’ reply that “the average plane of the phosphates [is] a bad reference to measure the variation of the depth of anchorage of individual amino acids.” As far as I understand, it is this measure that is shown in figures 3D, 5D, 7C, and 8D?*

**Authors – R2:** the key word in the sentence cited by the reviewer is “variation”: “the average plane of the phosphates [is] a bad reference to measure the **variation** of the depth of anchorage of individual amino acids.” This is because the z-coordinate of the average plane of the phosphates varies along simulation time, and therefore is not an ideal reference to follow the variation of the depth of insertion along a trajectory. On Figures 3D, 5D, 7C and 8D, we measure the depth of insertion on **single** **simulation snapshots**, in addition to providing electron density calculated over the trajectories of the production runs.

*(18) When discussing the Fig. 8 in the text, two numbers are given: 15+/-4% and 28+/-4%. How were these exactly calculated?*

**Authors - R1:**

Peak areas for the bands in each gel lane were measured using ImageJ. The percent of the protein bound by the liposomes was calculated from the area of the band in the pellet lane divided by the total area of the pellet and supernatant bands. This percent was then corrected to subtract the apparent percent bound observed in a negative control (run on the same gel) in which no liposome was added to a sample of the same protein. The measured percent binding from three independent runs (including different protein preps) were averaged and the uncertainty reported as the standard error of the mean. This is how we arrived at 15 ± 4% binding for wt and 28 ± 4% binding for the mutant. We also note that as stated in the Figure 9 caption, the percent bound was always higher for the mutant (by 10-17%) in each of the three independent measurements, giving an increase in binding of 13 ± 2% using standard error of the mean.

***Reviewer – R2:*** *What I still find confusing is that these numbers (15% and 28%) do not appear in Fig 9. If I now understand correctly, the number 23% in Fig 9 is the value for this particular experiment, and the corresponding value 15 ± 4% in the text is the value from all three repeats? And again 36% is for this particular experiment, and 28 ± 4% for all the three repeats? Maybe all the numbers for the three independent runs could be given in a table, so the reader could work out on their own, how these different numbers are connected?*

**Authors – R2:** we have now added the numbers in Table S5, and referred to that table in the caption of Figure 9.

*(21) Typos:*

*Across the manuscript, both ‘L1_αIII1i’ and ‘L1_αIII1’ are used. Maybe the ending ‘i’s could be consistently removed?*

Corrected

*Also, the characters ‘_’ and ‘-’ in protein names are being both used, i.e., there is for example both ‘Li_αΙΑ1’ and ‘Li-αΙΑ1’ as well as ‘L1_αIII1’ and ‘L1-αIII1’.*

Corrected

*p6: ‘Clustal Omega (35) was used to align multiple sequences ~~alignment~~.’*

Corrected

*p8: ‘The composition of the ~~eight~~* ***nine*** *simulated systems.’*

Corrected

*p9: ‘the distance between the hydrogen and the ~~donor~~* ***acceptor*** *of hydrogen bond atom should be below or equal to 2.4 Å’*

Corrected

*p15: ‘POPC:POPE bilayer. The hydrogen* ***bond*** *network between Li_αIA1 and the bilayer’ p19:*

*Missing citation: ’This result is consistent with the observation made by Cheng et al.* ***(27)*** *Indeed, when’*

Corrected

*Caption of Figure S12: ‘Minimum protein-bilayer distance in simulations of Li_αIA1, ~~Ll_αIII1~~, and WT St_βIB1 ~~and R44Y/S60Y~~ with a POPC:POPE (50:50) bilayer. Replica 1 is represented in orange and replica 2 in cyan.’*

Corrected
